# Bibliometric analysis of research on spinal cord injury and the gut microbiota

**DOI:** 10.3389/fmicb.2026.1852992

**Published:** 2026-08-06

**Authors:** Renkun Huang, Guohua Jiang, Xingyun Cai, Chuan Lu, Yuchang Gui, Jianwen Xu

**Affiliations:** Department of Rehabilitation Medicine, The First Affiliated Hospital of Guangxi Medical University, Nanning, Guangxi, China

**Keywords:** bibliometrics, gut microbiota, gut-spinal cord axis, probiotics, short-chain fatty acids, spinal cord injury

## Abstract

**Objective:**

To map the research landscape, knowledge base, hotspots, and emerging trends in studies of spinal cord injury (SCI) and the gut microbiota using bibliometric methods.

**Methods:**

English-language original articles and reviews on SCI and the gut microbiota published from January 1, 2000, to March 25, 2026 were retrieved from the Web of Science Core Collection (WoSCC) and Scopus. VOSviewer, CiteSpace, and Bibliometrix were used to analyze publication trends, collaboration networks, co-cited references, journals, authors, and keyword evolution.

**Results:**

A total of 4,857 publications were included, comprising 3,134 original articles and 1,723 reviews. Annual output increased markedly after 2019. China and the United States were the leading contributors, and Capital Medical University and Wenzhou Medical University were among the most productive institutions. Core journals included Spinal Cord, The Journal of Spinal Cord Medicine, Experimental Neurology, and Journal of Neurotrauma. Co-citation and keyword analyses indicated a shift in research attention from clinical phenotypes and complication management toward the gut-spinal cord axis, microbiota-derived metabolites, neuroinflammation, immune regulation, probiotics, short-chain fatty acids, and ferroptosis.

**Conclusion:**

Research on SCI and the gut microbiota has become a rapidly growing interdisciplinary field. Future studies should strengthen causal inference, mechanistic validation, multi-omics integration, and high-quality clinical translation of gut microbiota-targeted interventions.

## 1 Introduction

The spinal cord is an essential component of the central nervous system. It serves as a critical hub for bidirectional transmission of neural signals, maintenance of visceral homeostasis, and regulation of somatic motor function (Sun et al., 2021). Spinal cord injury (SCI) is structural and functional damage to the spinal cord caused by trauma, ischemia, degenerative disorders, and other factors, which may result in sensory, motor, and autonomic dysfunction below the level of injury. SCI is frequently accompanied by multisystem complications, including urinary tract infection, neurogenic bladder, and pressure ulcers, and is characterized clinically by a high disability rate, prolonged rehabilitation, and substantial social and healthcare burdens (Elliott et al., 2025). At present, clinical management of SCI remains primarily based on surgical decompression, symptomatic pharmacotherapy, rehabilitation training, and cell transplantation. Although these approaches may, to some extent, delay disease progression and improve patients’ quality of life (Hu et al., 2023), no definitive therapy is currently available to reverse irreversible spinal neuronal injury and achieve complete restoration of neurological function. Therefore, identifying novel regulatory mechanisms and potential therapeutic targets involved in the pathological progression of SCI has become a key scientific challenge in neuroscience and rehabilitation medicine.

In recent years, the bidirectional regulatory relationship between the gut microbiota and SCI has emerged as a major research focus at the intersection of neuroscience, rehabilitation medicine, microbiology, and immunology (Hamilton et al., 2024). The trillions of microorganisms residing in the human gastrointestinal tract constitute the gut microbiota, the most complex microbial ecosystem of the host. Dominated primarily by the phyla Firmicutes and Bacteroidetes, the gut microbiota plays indispensable roles in maintaining intestinal barrier integrity, regulating host immune homeostasis, and mediating nutrient metabolism (Chen et al., 2021). The composition and abundance of the gut microbiota are influenced by multiple factors, including host genetic background, dietary patterns, traumatic stress, and pharmacological interventions. Disruption of microbial homeostasis may contribute to the pathophysiological processes of the central nervous system through immune, metabolic, and neuroendocrine pathways (Neurath et al., 2025; Mincic et al., 2024). The gut-brain axis is a central pathway that mediates bidirectional communication between the central nervous system and the gut microecological environment, serving as a key intermediary through which the gut microbiota modulates neural repair and the progression of secondary injury after SCI (Ohara and Hsiao, 2025). Meanwhile, increasing attention has also been directed toward the direct regulatory role of the gut-spinal cord axis, whereby aberrant microbial signals may further exacerbate post-SCI neuroinflammatory cascades, disrupt the blood-spinal cord barrier, and promote neuronal death (Morimoto et al., 2023). Existing evidence indicates that the gut microbiota is involved in the entire course of SCI onset, progression, and repair through multiple pathways; the targeted modulation of gut microbial composition and function through probiotics, prebiotics, and fecal microbiota transplantation has become a promising therapeutic strategy for promoting neural repair after SCI (Xie et al., 2025; Jamali et al., 2025).

Bibliometrics is a quantitative, systematic research approach that uses mathematical and statistical methods to analyze literature in a specific field. Visualization techniques can reveal disciplinary development trajectories, collaboration networks, knowledge structures, and research hotspots, and they have been widely applied in medical research (Ninkov et al., 2022). Over the past 26 years, research on SCI and the gut microbiota has grown rapidly. However, to date, no systematic evaluation of the global research landscape, hotspot evolution, and emerging trends in this field has been conducted using multi-database, multidimensional bibliometric methods. Accordingly, the present study employed VOSviewer, CiteSpace, and Bibliometrix to conduct a comprehensive bibliometric analysis of relevant publications indexed in the Web of Science Core Collection (WoSCC) and Scopus databases from January 2000 to March 2026. This study aimed to delineate publication trends, national and institutional collaboration networks, distributions of core authors and journals, co-citation-based knowledge foundations, and the evolution of keyword hotspots, thereby providing a comprehensive overview of the current status, developmental patterns, and frontier dynamics of this field, as well as a scientific reference for subsequent mechanistic research, target discovery, and clinical translation in SCI-related neural repair.

## 2 Materials and methods

### 2.1 Data sources

To ensure the comprehensiveness, authority, and completeness of the study data, two internationally recognized core academic databases were selected for literature retrieval and collection: WoSCC and Scopus. WoSCC is one of the most widely used core databases in academic publishing and citation analysis, covering high-quality peer-reviewed journals across multiple disciplines. Its standardized metadata and citation indexing system is highly compatible with mainstream bibliometric analysis software. It is frequently used to analyze academic development trends, scientific collaboration networks, and research hotspots (Birkle et al., 2020). Scopus, launched by Elsevier in 2004, is currently one of the most widely used comprehensive abstract and citation databases worldwide. It indexes a large volume of high-quality interdisciplinary academic publications. It can effectively compensate for the limited coverage of a single database, thereby enhancing the breadth and completeness of research data (Baas et al., 2020).

PubMed was not included in the present study, primarily because its bibliographic records lack several key metadata fields required for standardized bibliometric analysis. Unlike WoSCC, PubMed does not provide standardized citation counts, reference lists, or other citation linkage information. Moreover, PubMed uses a distinct literature classification system and indexes numerous clinical trial-related records, making it difficult to precisely restrict the search scope to original research articles and review articles. Furthermore, PubMed records often lack important information such as authors’ institutional affiliations and countries/regions. Mainstream bibliometric analysis tools are currently optimized for the data structures of WoSCC and Scopus and are not well adapted to the PubMed format. To avoid inconsistencies and bias arising from incomplete metadata, only Scopus and WoSCC were included in this study, as both provide standardized datasets with comprehensive coverage and high compatibility with bibliometric analysis software.

All literature retrieval procedures were completed on the same day to minimize retrieval bias from daily database updates and to ensure data consistency. The retrieval period spanned from January 1, 2000 to March 25, 2026. The entire bibliometric workflow was conducted in accordance with the data extraction and analysis principles of the Preferred Reporting Items for Systematic Reviews and Meta-Analyses 2020 (PRISMA 2020) statement, and the completed PRISMA 2020 checklist is provided as Supplementary Appendix S1.

The inclusion criteria were as follows: (1) original research articles or review articles; and (2) publications in English. The exclusion criteria were as follows: (1) non-English publications; (2) preprints, conference papers, conference abstracts, book chapters, letters to the editor, and retracted publications; and (3) publications unrelated to SCI and gut microbiota-related regulatory mechanisms.

### 2.2 Search strategy and screening process

**Figure 1:**
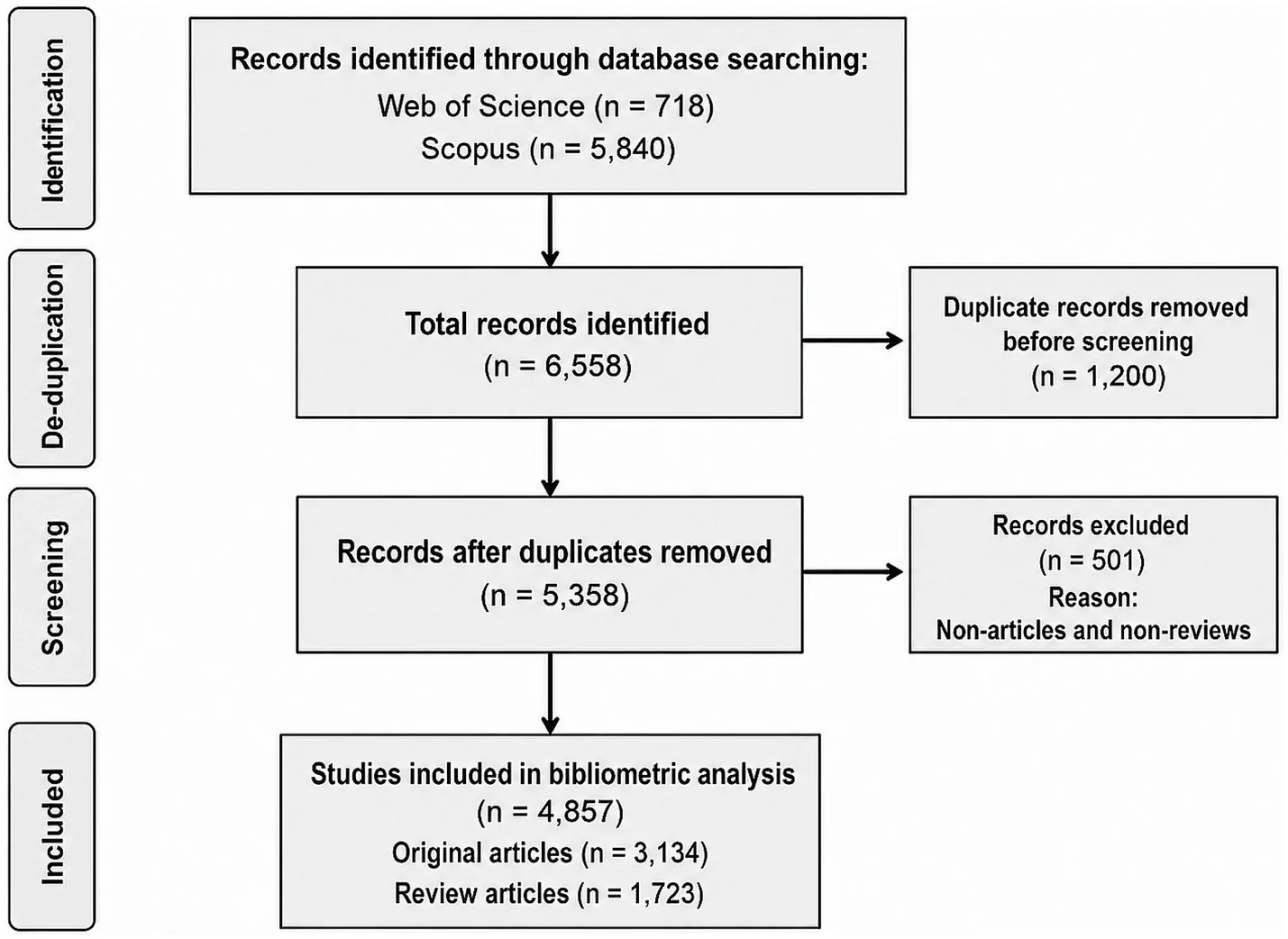
Flowchart of literature screening and data processing for bibliometric analysis.

A two-stage screening procedure was conducted independently by two researchers (Renkun Huang and Guohua Jiang). First, titles and abstracts were screened to exclude publications that were clearly inconsistent with the inclusion criteria. Subsequently, a full-text review was performed for the remaining studies to confirm their relevance to the research topic. In cases of disagreement between the two reviewers, consensus was reached through discussion with a third senior investigator (Xingyun Cai), who also made the final determination. The complete search strategy is provided in Supplementary Appendix S2, and the literature screening process is shown in Figure 1.

### 2.3 Data standardization

In this study, VOSviewer was used to extract the countries/regions and keywords of the included publications. After data cleaning, term standardization was performed using the “thesaurus file” function to merge and correct terms. The specific procedures were as follows:

1. Standardization of country/region names:

Internationally accepted standard names were used to normalize country/region names. For example, “People’s Republic of China” was standardized as “China,” “United States of America” as “United States,” and “England” as “United Kingdom.” Furthermore, territorial affiliations with sovereign states were explicitly defined. For instance, Taiwan, the Hong Kong Special Administrative Region, and the Macao Special Administrative Region of China were all classified under “China,” whereas Scotland and Wales were grouped under “United Kingdom,” to ensure consistency and rigor in geographic information.

1. Keyword standardization:

- Merging of synonymous keywords: Keywords with identical core meanings were unified. For example, spinal cord lesion and “SCI” were merged into “SCI,” and “intestinal microbiota” was standardized as “gut microbiota.”
- Standardization of variant expressions: Keywords with differing written forms but equivalent meanings were normalized. For example, “gut-brain axis” was standardized as “gut brain axis,” and “anti-bacterial agents” as “anti-bacterial agent.”
- Standardization of singular and plural forms: Plural noun forms were converted into singular forms; for example, “animals” was standardized as “animal,” and “humans” as “human.”

### 2.4 Visualization tools and analysis

Multiple professional software tools were used to conduct bibliometric analysis and data visualization in the field of SCI and the gut microbiota. VOSviewer version 1.6.20 was used to construct visual knowledge maps of collaboration networks among countries/regions, research institutions, core authors, and academic journals, and to generate keyword density maps, keyword co-occurrence networks, and reference co-citation networks with cluster analysis. CiteSpace version 6.4. R1 was used to construct temporal network models using time-slicing techniques, generate dual-map overlays of journals and timeline views of reference co-citation clusters, and detect burst strengths of keywords and highly cited references. RStudio with the Bibliometrix 5.0 package was used for statistical analysis of core bibliometric data, calculation of international collaboration characteristics, and to generate keyword strategic diagrams based on the centrality-density model and thematic trend evolution maps over time. Microsoft Office Excel 2019 was used for data collation and cleaning, preparation of summary tables for core indicators, and plotting of annual publication trends.

## 3 Results

### 3.1 Annual publication trends

**Figure 2:**
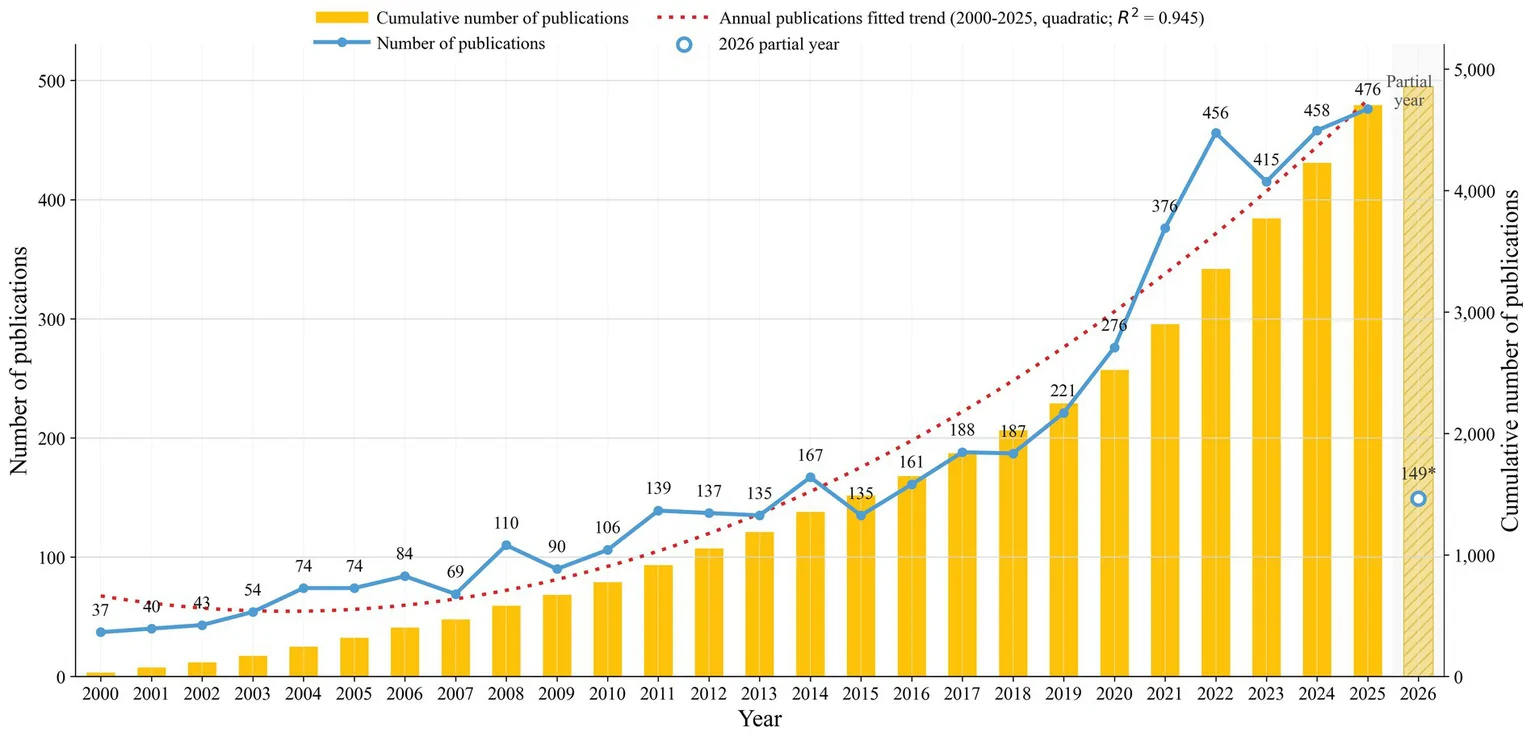
Annual publication output and cumulative publication trends in research on spinal cord injury and the gut microbiota. The blue line indicates annual publication output, the yellow bars indicate cumulative publication output, and the red dashed line indicates the fitted trend based on annual publication counts from 2000 to 2025 using a quadratic model (*R*^2^ = 0.945). The 2026 data represent a partial year because the search was conducted on March 25, 2026; therefore, the 2026 data point was marked separately, not connected to the annual publication line, and excluded from the fitted trend.

A total of 4,857 publications related to SCI and the gut microbiota were included in this study, comprising 3,134 original articles and 1,723 reviews. Figure 2 illustrates the annual and cumulative publication trends in this field from 2000 to 2026. Overall, the annual number of publications showed a slow upward trend during the early stage, followed by a marked acceleration after 2019. The annual output increased from 221 publications in 2019 to 276 in 2020 and 376 in 2021, then remained at a relatively high level, with with 456 in 2022, 415 in 2023, and 458 in 2024, reaching a peak of 476 publications in 2025. To avoid overinterpretation of cumulative growth, the fitted curve in Figure 2 was generated based on annual publication counts from 2000 to 2025 rather than cumulative publication counts, showing a good fit to the overall increasing trend (R^2^ = 0.945). The cumulative number of publications also increased steadily during the same period, indicating that research at the intersection of SCI and the gut microbiota has continued to develop over the past two decades.

### 3.2 Distribution of countries and institutions

**Figure 3:**
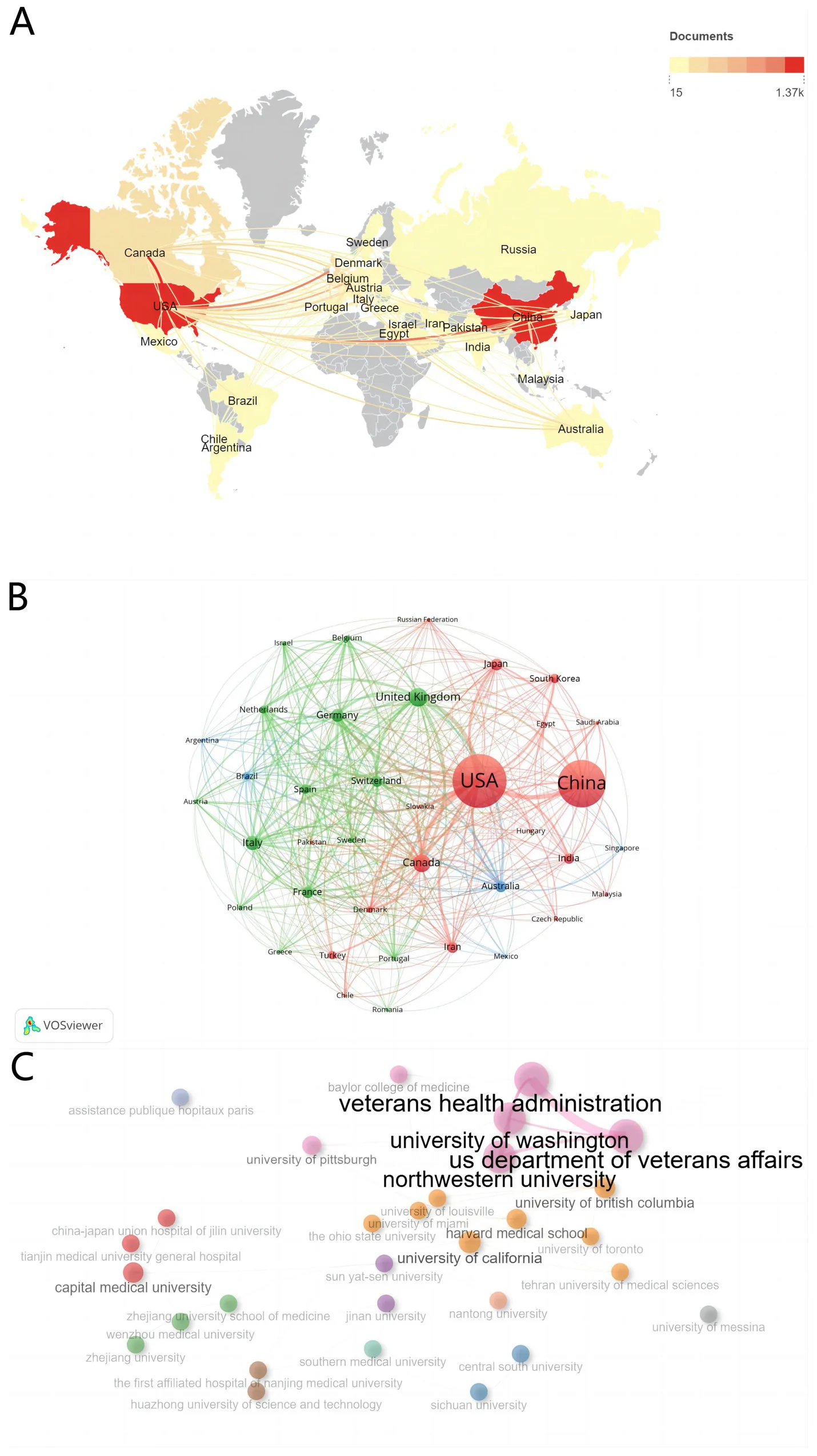
Visualization of country and institutional participation in research on spinal cord injury and the gut microbiota. **(A)** Global geographical distribution map of publication output generated by VOSviewer in combination with Scimago Graphica; Color intensity indicates publication volume. **(B)** Country collaboration network generated by VOSviewer. Nodes represent countries, node size is positively correlated with publication output, and line thickness indicates collaboration strength. **(C)** Institutional collaboration network generated by VOSviewer. Nodes represent research institutions; node size is proportional to publication output; links indicate collaborative relationships; and line thickness reflects collaboration strength.

From 2000 to 2026, a total of 82 countries conducted research on SCI and the gut microbiota. The geographical distribution of research output (Figure 3A) showed marked regional clustering, with Asia, North America, and Europe serving as the major research centers in this field.

The independent and collaborative publication outputs of the top 10 countries are presented in Table 1. China ranked first globally with 1,303 publications, accounting for 26.8% of the total output and markedly surpassing all other countries. This advantage may be related to China’s large population base and substantial SCI patient population, with growing clinical demand strongly driving basic research and scientific output in this field. The United States ranked second with 1,243 publications (25.6%), followed by Canada (180 publications, 3.7%), the United Kingdom (176 publications, 3.6%), and Italy (172 publications, 3.5%). Together, these five countries accounted for more than 63% of total research output and were the principal driving forces behind the development of the field.

**Table 1:** Top 10 countries by publication output and independent or collaborative publications.

Rank	Country	Articles	Articles %	SCP	MCP	MCP %
1	China	1,303	26.8	1,292	11	0.8
2	United States	1,243	25.6	1,219	24	1.9
3	Canada	180	3.7	171	9	5
4	United Kingdom	176	3.6	161	15	8.5
5	Italy	172	3.5	165	7	4.1
6	Japan	128	2.6	126	2	1.6
7	Iran	123	2.5	118	5	4.1
8	India	112	2.3	110	2	1.8
9	Germany	96	2	92	4	4.2
10	Australia	91	1.9	82	9	9.9

The international collaboration network (Figure 3B) shows that a broad global academic collaboration network has been established. In the map, nodes represent countries, node size is proportional to publication output, and line thickness reflects the strength of collaboration between countries. Among them, China and the United States exhibited the closest collaboration and jointly formed the central hub of international cooperation in this field.

VOSviewer analysis showed that 5,035 institutions contributed publications on SCI and the gut microbiota. The top 10 institutions by publication output are listed in Table 2. Capital Medical University ranked first with 261 publications, highlighting its outstanding research strength in the interdisciplinary area of SCI and gut microecology. It was followed by Wenzhou Medical University (215 publications), the University of British Columbia (208 publications), the University of California (173 publications), and Sun Yat-sen University (170 publications), which together constituted the core institutional research force in this field.

**Table 2:** Top 10 research institutions by publication output.

Rank	Institution	Articles	Country
1	Capital Medical University	261	China
2	Wenzhou Medical University	215	China
3	University of British Columbia	208	Canada
4	University of California	173	United States
5	Sun Yat-Sen University	170	China
6	Sichuan University	164	China
7	University of Messina	138	Italy
8	Southern Medical University	133	China
9	Tehran University of Medical Sciences	132	Iran
10	Central South University	123	China

The institutional collaboration network (Figure 3C) indicates that Chinese universities have formed a closely connected academic collaboration framework, with Capital Medical University, Wenzhou Medical University, Sun Yat-sen University, and Sichuan University occupying central positions in domestic cooperation. International academic collaboration was mainly concentrated between leading Chinese universities and well-established overseas institutions. Representative examples include close cooperation between major Chinese universities and the University of British Columbia and the University of California system, as well as the mature collaborative network formed among the U. S. Department of Veterans Affairs, the Veterans Health Administration, Harvard Medical School, and Northwestern University.

### 3.3 Author distribution and collaboration network

Between 2000 and 2026, a total of 23,729 scholars participated in research on SCI and the gut microbiota, forming a collaborative research landscape involving several core authors and scholars from different countries and regions. Table 3 lists the most productive authors in this field. Evans, Charlesnika T ranked first with 50 publications, making her the most productive author in this field. Burns, Stephen P ranked second with 29 publications, followed by Popovich, Phillip G with 28 publications. Esposito, Emanuela, Weaver, Frances M, Cuzzocrea, Salvatore, and Fehlings, Michael G published 24, 23, 21, and 20 papers, respectively, showing relatively stable research productivity. Among these authors, Popovich, Phillip G had the highest within-dataset citation count, reaching 2,156 citations. Cuzzocrea, Salvatore, Esposito, Emanuela, and Fehlings, Michael G also had high within-dataset citation counts, with 1,403, 1,332, and 1,220 citations, respectively, suggesting that their research outputs have exerted relatively strong academic influence in this field.

**Table 3:** Most productive authors and their within-dataset citation counts.

Rank	Author	Documents	Citations
1	Evans, Charlesnika T	50	936
2	Burns, Stephen P	29	386
3	Popovich, Phillip G	28	2,156
4	Esposito, Emanuela	24	1,332
5	Weaver, Frances M	23	340
6	Cuzzocrea, Salvatore	21	1,403
7	Fehlings, Michael G	20	1,220
8	Pannek, Jürgen	19	646
9	Suda, Katie J	19	298
10	Mei, Xifan	18	524
11	Yu, Yan	18	502
12	Fitzpatrick, Margaret A	18	159

**Figure 4:**
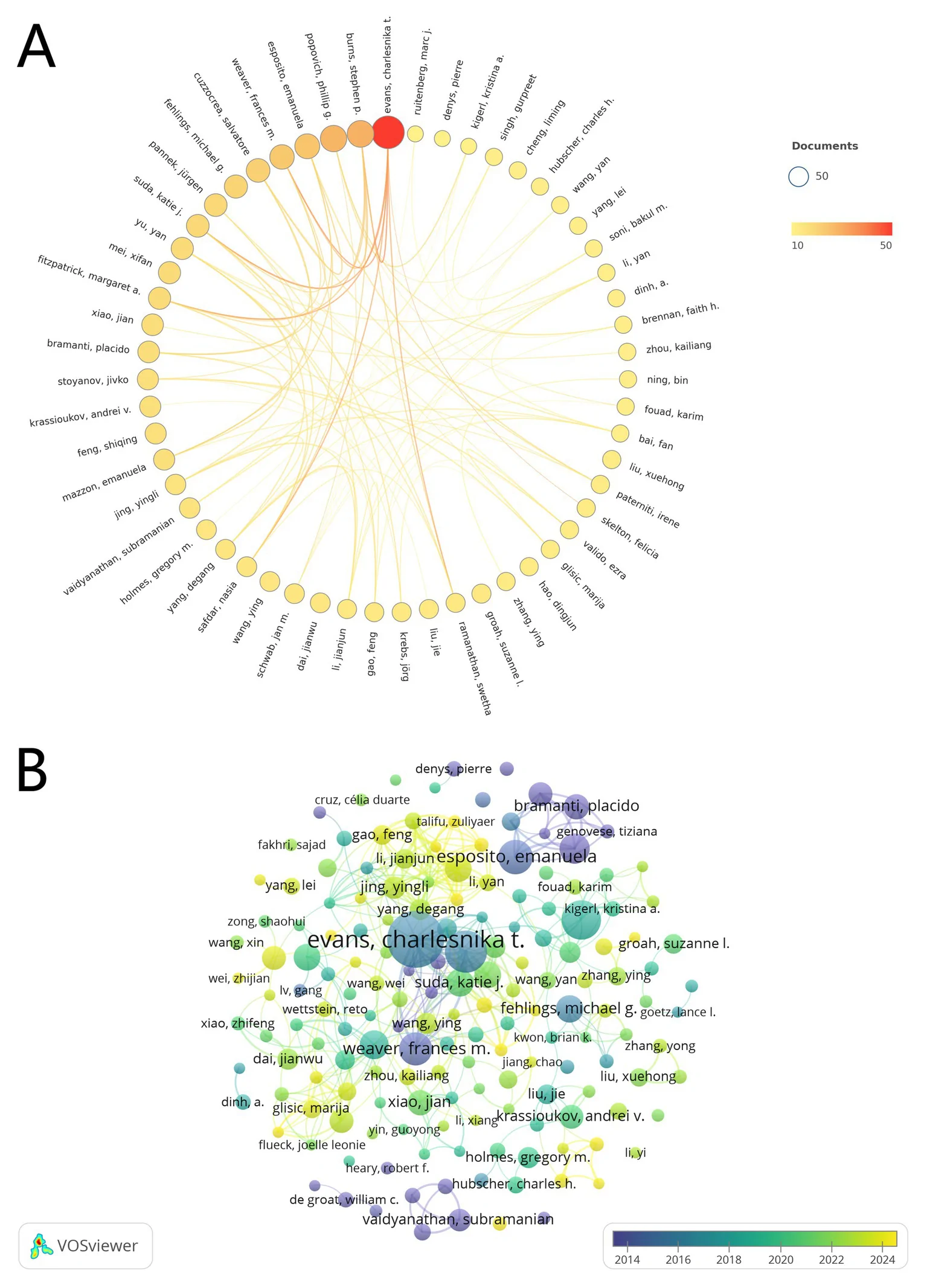
Author collaboration network in research on spinal cord injury and the gut microbiota. **(A)** Author collaboration network constructed using VOSviewer. Nodes represent contributing scholars, node size is positively correlated with publication output, links indicate collaborative relationships between scholars; line thickness reflects collaboration strength; and different colors denote different collaboration clusters. **(B)** Temporal evolution map of author collaboration generated by VOSviewer. Nodes represent scholars, and node colors gradually transition from blue to yellow, with more yellow tones indicating later periods of collaboration.

In the author collaboration network (Figure 4A), node size is proportional to publication output, and links represent collaborative relationships between scholars. The temporal evolution map of author collaboration (Figure 4B) further illustrates the evolution of collaboration patterns in this field, with node colors gradually shifting from blue to yellow to represent earlier to more recent periods of collaboration. The visualization shows several relatively stable author collaboration groups. The group centered on Evans, Charlesnika T, Burns, Stephen P, Weaver, Frances M, and Suda, Katie J showed close collaborative relationships and represented an important author cluster in this field. The group involving Esposito, Emanuela, Bramanti, Placido, Cuzzocrea, Salvatore, and related authors formed another visible collaboration cluster. Furthermore, Chinese authors such as Li, Jianjun, Gao, Feng, Jing, Yingli, Yu, Yan, and their collaborators have shown more recent collaborative activity, reflecting the increasing research contribution from China in this field. Overall, although collaborative links have been established among several core authors, connections between different clusters remain relatively limited, and some highly productive or highly cited authors are located at the periphery of the network. These findings suggest that collaboration in this field remains primarily concentrated within specific research groups or regional networks, and that deeper cross-team and cross-regional collaboration requires further strengthening.

### 3.4 Journal analysis

Research in this field has appeared in 1,462 journals, primarily in leading journals across neuroscience, rehabilitation medicine, microbiology, clinical medicine, and related disciplines. Table 4 lists the top 10 journals by publication output. Spinal Cord ranked first with 163 publications and served as the principal publication platform in this field, followed by The Journal of Spinal Cord Medicine with 113 publications. Experimental Neurology (80 publications), Journal of Neurotrauma (76 publications), Neural Regeneration Research (73 publications), and International Journal of Molecular Sciences (72 publications) ranked third through sixth, respectively. These 10 journals collectively published 740 articles, accounting for 15.2% of the total output and forming the core journal cluster in this field.

**Table 4:** Top 10 journals by publication output.

Rank	Journal	Publications	IF	JCR
1	Spinal Cord	163	2.2	Q1
2	Journal of Spinal Cord Medicine	113	1.5	Q2
3	Experimental Neurology	80	4.2	Q1
4	Journal of Neurotrauma	76	3.8	Q1
5	Neural Regeneration Research	73	6.7	Q1
6	International Journal of Molecular Sciences	72	4.9	Q1
7	Molecular Neurobiology	46	4.3	Q1
8	Frontiers in Immunology	44	5.9	Q1
9	Neurourology and Urodynamics	38	1.9	Q2
10	Plos One	35	2.6	Q1

Regarding journal impact, 7 of the top 10 journals were classified as JCR Q1 journals. Among them, Neural Regeneration Research had the highest impact factor (6.7) and primarily published interdisciplinary studies on neural regeneration and SCI repair. Journals such as Frontiers in Immunology (IF = 5.9), International Journal of Molecular Sciences (IF = 4.9), and Molecular Neurobiology (IF = 4.3) also demonstrated substantial academic influence, further underscoring the scholarly authority of the core journals in this field.

**Figure 5:**
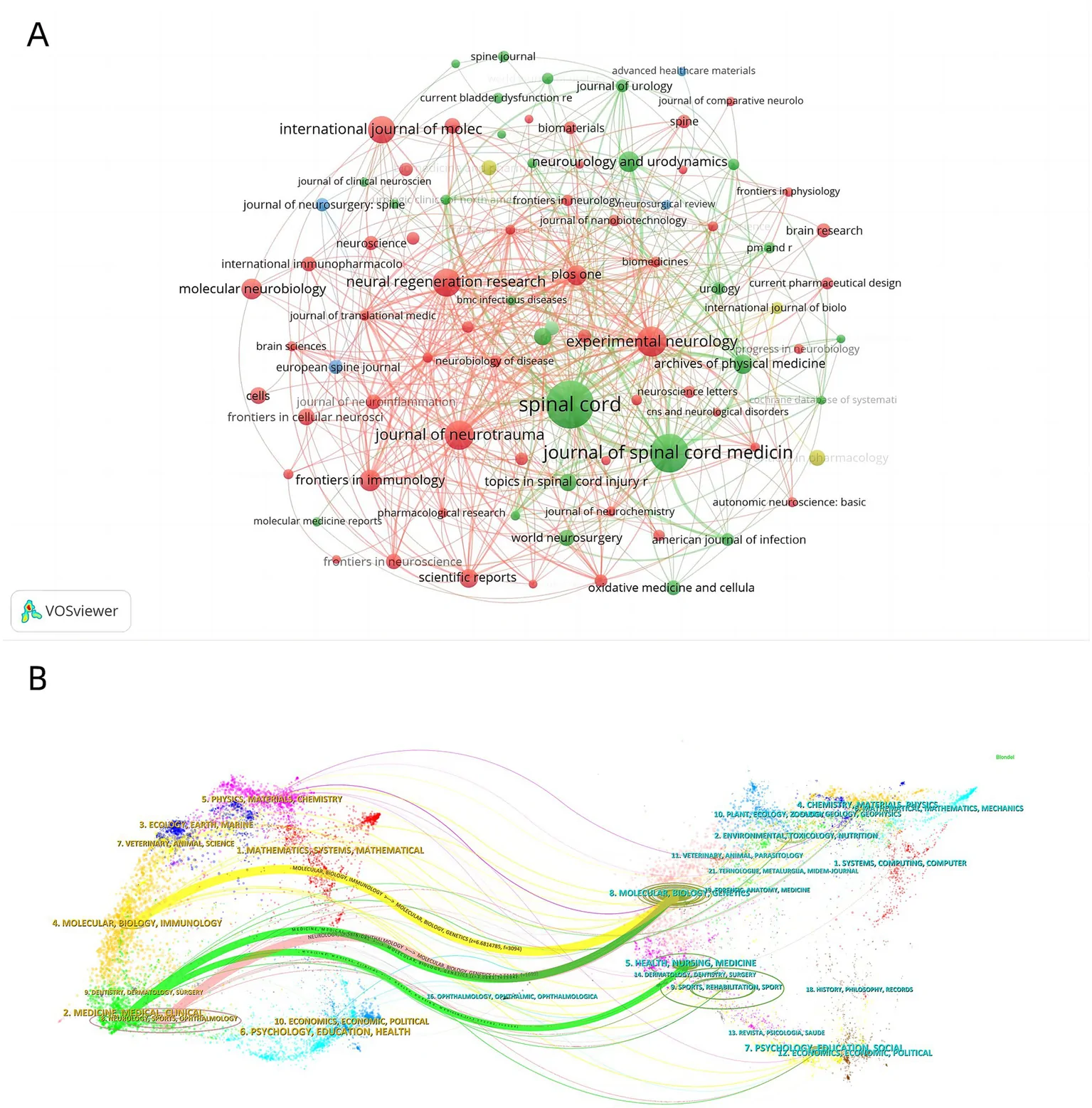
Journal co-occurrence and disciplinary distribution in research on spinal cord injury and the gut microbiota. **(A)** Journal co-occurrence network constructed using VOSviewer. Nodes represent journals, node size is positively correlated with journal publication output, and links indicate the degree of thematic association between journals. **(B)** Dual-map overlay of journals generated by CiteSpace. The left side shows disciplinary clusters of citing journals, representing the research frontiers, whereas the right side shows disciplinary clusters of cited journals, representing the knowledge base. Different colors denote different academic disciplines, and the lines indicate citation flow across disciplines.

The journal co-occurrence network map (Figure 5A) showed that several major thematic clusters have emerged in this field. The green cluster, centered on Spinal Cord and The Journal of Spinal Cord Medicine, primarily publishes studies in the clinical management and rehabilitation of SCI. The red cluster, centered on Experimental Neurology and Journal of Neurotrauma, encompasses basic research on the pathological mechanisms of neural injury and neural regeneration. The branch clusters centered on Frontiers in Immunology and International Journal of Molecular Sciences focus on interdisciplinary research involving immune regulation by the gut microbiota, molecular mechanisms, and SCI.

The dual-map overlay of journals (Figure 5B) shows that the research frontiers in this field are primarily concentrated in applied disciplines such as molecular biology, immunology, clinical medicine, and rehabilitation medicine, whereas the knowledge base is derived from fundamental disciplines including molecular biology, genetics, systems science, physics, and materials science. The core citation pathways suggest that basic disciplines provide important support for applied research in the interdisciplinary field of SCI and gut microecology; however, the linkage between clinical rehabilitation disciplines and the frontiers of basic research still requires further strengthening. The multidisciplinary nature of the literature included in this study further supports this conclusion.

### 3.5 Co-citation analysis

Using VOSviewer, this study conducted a co-citation analysis of the 4,857 included publications to identify highly influential literature in the field of SCI and the gut microbiota, thereby providing a basis for interpreting research hotspots and developmental trends. Notably, the citation counts reported in this section and in Table 5 refer to within-dataset citation or co-citation counts generated from the 4,857 included publications, rather than global citation counts from Web of Science, Scopus, or other citation databases. Table 5 lists the top 10 most frequently cited publications in the included dataset, including 8 original studies and 2 reviews.

**Table 5:** Top 10 most frequently cited publications within the included dataset.

Rank	Author	Journal	Title	Total citations
1	Kigerl et al. (2016)	The Journal of Experimental Medicine	Gut dysbiosis impairs recovery after spinal cord injury	105
2	Basso et al. (1995)	Journal of Neurotrauma	A sensitive and reliable locomotor rating scale for open field testing in rats	101
3	McDonald and Sadowsky (2002)	Lancet	Spinal-cord injury	79
4	Gungor et al. (2016)	PLoS One	Intestinal microbiota in patients with spinal cord Injury	73
5	Zhang et al. (2018)	Journal of Translational Medicine	Gut microbiota dysbiosis in men with chronic traumatic complete spinal cord injury	59
6	(Beck et al. (2010))	Brain	Quantitative analysis of cellular inflammation after traumatic spinal cord injury: evidence for a multiphasic inflammatory response in the acute to chronic environment	55
7	Basso et al. (2006)	Journal of Neurotrauma	Basso mouse scale for locomotion detects differences in recovery after spinal cord injury in five common mouse strains	53
8	O'Connor et al. (2018)	Journal of Neurotrauma	Investigation of microbiota alterations and intestinal inflammation post-spinal cord injury in rat model	52
9	Esclarín De Ruz et al. (2000)	The Journal of Urology	Epidemiology and risk factors for urinary tract infection in patients with spinal cord injury	51
10	Orr and Gensel (2018)	Neurotherapeutics	Spinal cord injury scarring and inflammation: therapies targeting glial and inflammatory responses	49

The study by Kigerl KA, published in The Journal of Experimental Medicine in 2016, ranked first with 105 within-dataset citations/co-citations. This study was the first to demonstrate that gut microbiota dysbiosis can significantly impair neurological recovery after SCI and represents a landmark publication that links gut microecology with SCI research, thereby establishing a core theoretical foundation for this field. The study by Basso DM, published in the Journal of Neurotrauma in 1995, ranked second with 101 within-dataset citations/co-citations, followed by the review by McDonald and Sadowsky, published in the Lancet in 2002 with 79 within-dataset citations/co-citations. The former established the Basso scale, the gold-standard open-field locomotor assessment for rats with SCI, thereby providing a standardized evaluation system for animal studies in this field. The latter systematically summarized the pathological mechanisms, clinical diagnosis and treatment, and research progress of SCI and remains a classic seminal review in this area. Notably, the 2016 study by the Kigerl group was the first to provide key experimental evidence supporting the involvement of gut microbiota in SCI repair, identifying “bidirectional regulation of the gut-spinal cord axis” and “microbiota-immune-neural repair mechanisms” as central research hotspots, and further indicating that “targeted gut microbiota intervention” would become an important new direction in translational research on SCI repair.

**Figure 6:**
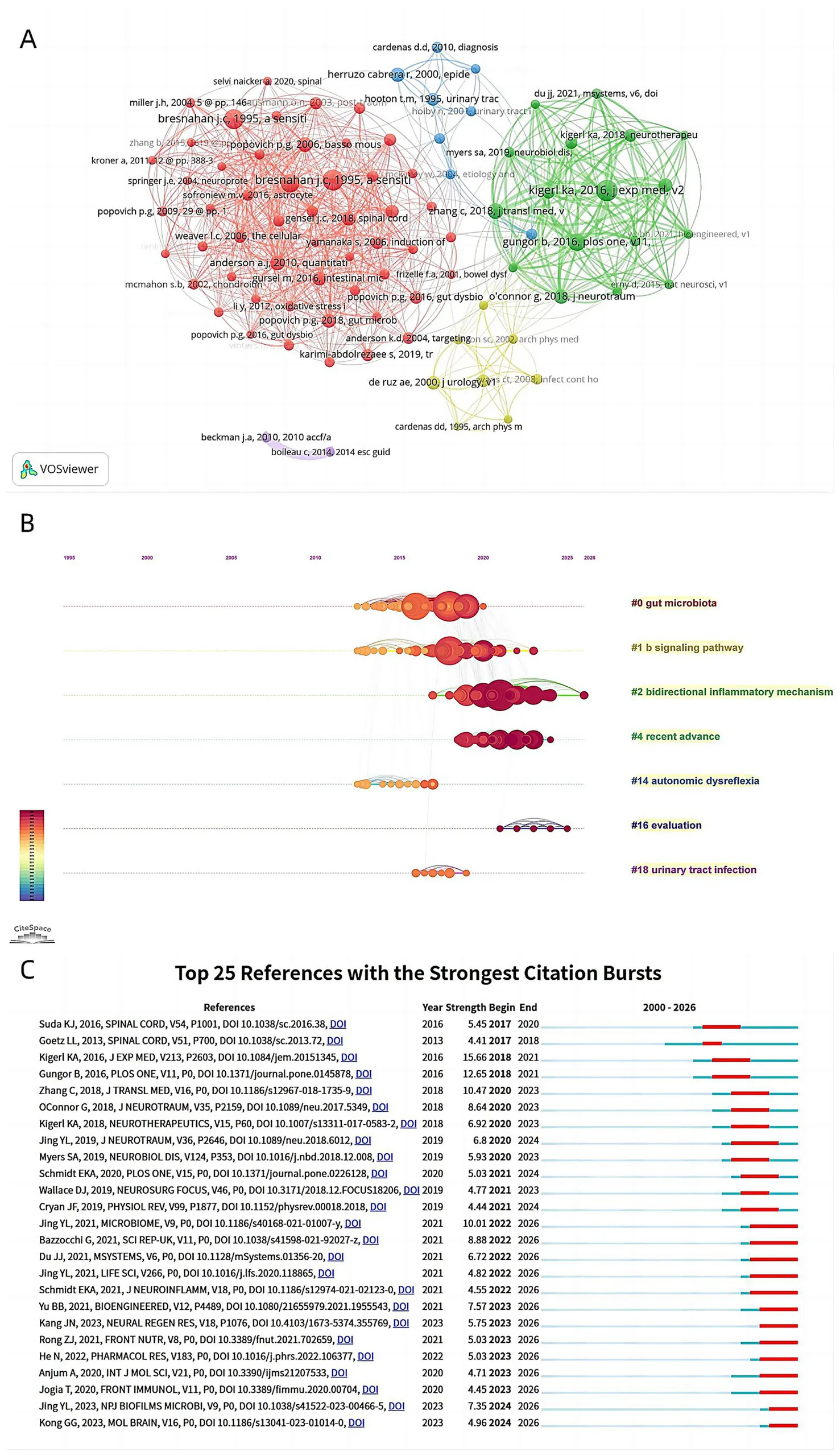
Co-citation and burst analysis of the literature on spinal cord injury and the gut microbiota. **(A)** Literature co-citation network constructed using VOSviewer. Nodes represent individual publications, node size is positively correlated with citation frequency, links indicate co-citation relationships between publications, and different colors denote different co-citation clusters. **(B)** Timeline view of literature co-citation clusters generated by CiteSpace. The horizontal axis represents time; nodes represent publications, node size is proportional to citation frequency; different colors correspond to different cluster themes; and the vertical arrangement clearly illustrates the temporal evolution of each research cluster. **(C)** Top 25 references with the strongest citation bursts from 2000 to 2026. Red line segments indicate the time intervals during which each reference exhibited a citation burst, and the values in the “Strength” column represent burst intensity, reflecting the leading influence of each reference on the development of the field during the corresponding period.

VOSviewer was used to construct a co-citation network of highly cited references (Figure 6A). In the network, node size was positively correlated with within-dataset citation/co-citation frequency, and link strength reflected the degree of co-citation between references. Based on co-citation relationships, the network was divided into multiple clusters. The red cluster, centered on “Basso DM, 1995, Journal of Neurotrauma” and “McDonald and Sadowsky, 2002, Lancet,” reflects the concentration of foundational evaluation systems and core research on pathological mechanisms in SCI. The green cluster, based on a series of studies by Kigerl KA (2016, 2018), Gungor B (2016), and Zhang C (2018), represents a sustained academic trajectory focused on the intersection of the gut microbiota and SCI and constitutes an emerging core research cluster in this field. The blue cluster includes studies by Beck KD (2010, Brain) and Orr MB (2018, Neurotherapeutics), revealing a broad relational network centered on post-SCI inflammatory responses and scar repair mechanisms.

CiteSpace was further used to conduct co-citation cluster analysis and burst detection. The cluster timeline map (Figure 6B) showed that the co-citation network could be divided into several core clusters. Nodes are distributed along a horizontal timeline corresponding to the citation time points of the references, and node size is positively correlated with within-dataset co-citation frequency, clearly illustrating the temporal evolution of research themes. Between 1995 and 2010, research hotspots were concentrated in clinical foundations and complication management, including “autonomic dysreflexia” (#14) and “urinary tract infection” (#18). Between 2010 and 2016, core themes related to pathological mechanisms, such as “bidirectional inflammatory mechanism” (#2) and “b signaling pathways” (#1), gradually became the focus of research. Since 2016, themes related to the “gut microbiota” (#0) have become increasingly prominent and have emerged as the most central hotspot in this field, reflecting the rapid transition of this area toward an interdisciplinary direction integrating neuroscience, rehabilitation medicine, and microbiology.

Burst detection analysis (Figure 6C) identified the top 25 references ranked by burst strength, with the burst strengths of the core references all exceeding 4.4. Burst strength reflects the intensity of a sudden increase in citations within the included dataset during a specific period and should not be interpreted as a global citation count. The 2016 study by Kigerl KA in The Journal of Experimental Medicine exhibited the highest burst strength (15.66) during 2018–2021. This study focused on the regulatory mechanisms by which gut microbiota dysbiosis affects post-SCI repair and represents the foundational study that has driven the explosive growth of this interdisciplinary field. The study by Gungor B, published in PLoS One in 2016, reported a burst strength of 12.65 and remained active through 2021. The paper by Zhang C, published in Journal of Translational Medicine in 2018, had a burst strength of 10.47 and remained active through 2023. The study by the Jing YL group published in Microbiome in 2021 had a burst strength of 10.01 and persisted through 2026. These studies, focusing on the bidirectional regulatory mechanisms between the gut microbiota and SCI as well as clinical population characteristics, strongly corroborate the trends revealed by the cluster timeline and confirm that gut microbiota-related research constitutes the current and future core frontier of this field.

### 3.6 Keyword and thematic term analysis

#### 3.6.1 Keyword analysis

Keywords are essential for identifying the core themes of research articles. By analyzing keyword frequency co-occurrence relationships and temporal evolution research hotspots and developmental trends in a given field can be accurately identified. In this study VOSviewer was used to construct and visualize the keyword co-occurrence network while Bibliometrix was used to generate thematic strategic diagrams and analyze the temporal evolution of keywords. The keyword occurrence counts and total link strength values reported in Table 6 are network-based bibliometric indicators derived from the 4,857 included publications. Specifically occurrences indicate keyword frequency within the included dataset whereas total link strength reflects the intensity of co-occurrence relationships among keywords in the VOSviewer network. These values should not be interpreted as citation counts

**Table 6:** Top 20 keywords and total link strength in the keyword co-occurrence network.

Rank	Keyword	Occurrences	Total link strength
1	spinal cord injury	4,198	73,237
2	human	3,132	52,802
3	gut microbiota	2,597	53,006
4	nonhuman	2,359	50,142
5	chain fatty acid	2,230	39,674
6	men	1846	37,302
7	gut-brain axis	1721	39,554
8	neuroinflammation	1,580	36,667
9	probiotics	1,523	24,269
10	Women	1,505	31,383
11	adult	1,415	27,906
12	animal	1,380	33,403
13	ferroptosis	1,175	20,996
14	animal experiment	1,134	28,802
15	animal model	1,081	27,507
16	metabolism	1,021	25,389
17	animal tissue	911	23,982
18	rat	864	20,900
19	inflammation	829	17,616
20	antibiotic agent	722	12,367

**Figure 7:**
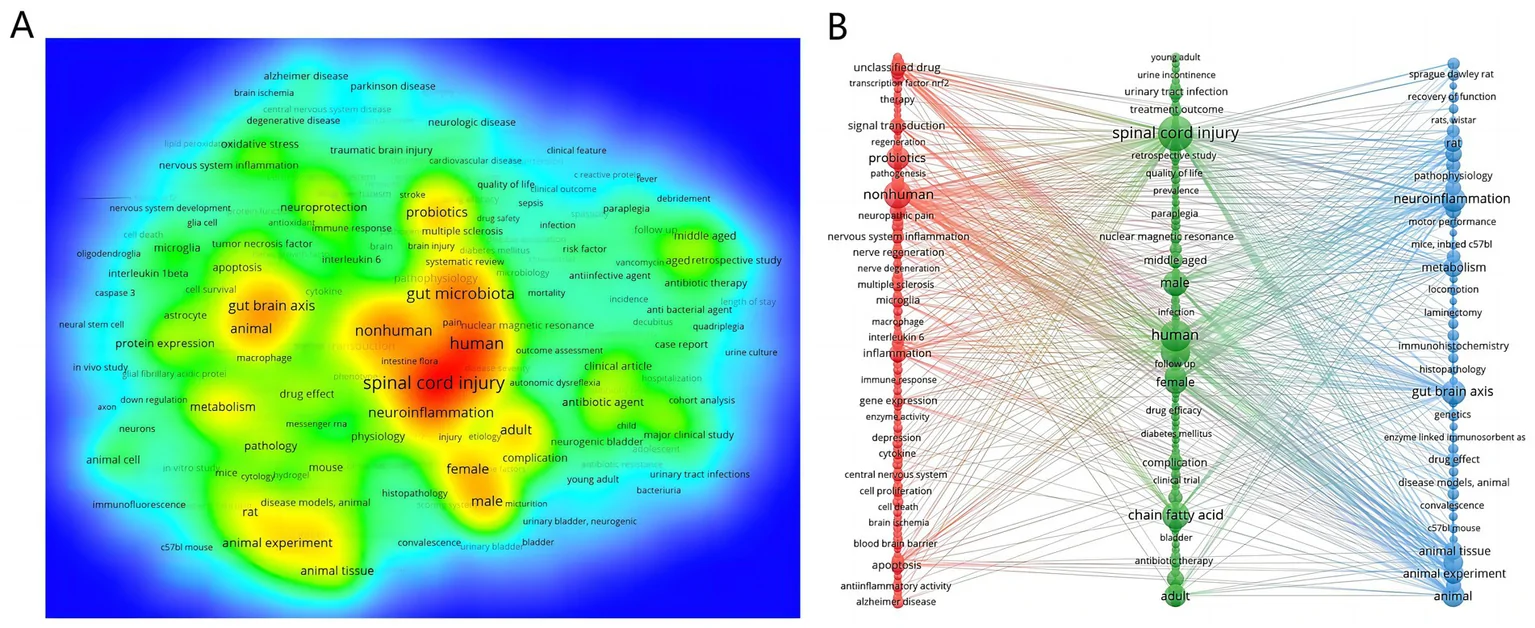
Keyword co-occurrence analysis in research on spinal cord injury and the gut microbiota. **(A)** Keyword density heat map generated by VOSviewer. The color gradient from blue to red indicates increasing keyword frequency, and font size is positively correlated with keyword occurrence. **(B)** Keyword clustering network generated by VOSviewer. Nodes represent keywords; node size is proportional to keyword occurrence frequency; links indicate co-occurrence relationships between keywords; and different colors denote different research theme clusters.

The keyword density map (Figure 7A) showed that “SCI” was the core theme in this field. Surrounding it, keywords such as “gut microbiota,” “human,” “nonhuman,” and “neuroinflammation” formed dense clusters, clearly reflecting the distribution of research priorities. The ranking of high-frequency keywords (Table 6) showed that “SCI” ranked first, with 4,198 occurrences and a total link strength of 73,237 within the keyword co-occurrence network, consistently maintaining its central thematic position in the field. Keywords such as “human,” “gut microbiota,” “nonhuman,” and “short-chain fatty acids” each occurred more than 2,000 times. They ranked second through fifth, highlighting a balanced emphasis on both clinical and basic research and a high level of attention directed toward gut microbiota-related mechanisms as a core research focus. Furthermore, keywords such as “gut-brain axis,” “neuroinflammation,” “probiotics,” “ferroptosis,” and “metabolism” each occurred more than 1,000 times, further indicating that post-SCI inflammatory regulation, gut-neural axis interactions, and mechanisms of secondary injury are key research directions in this field. However, these high-frequency keywords reflect research attention and thematic concentration rather than direct evidence of mechanistic importance, causality, or clinical efficacy.

The keyword co-occurrence network (Figure 7B) formed three closely connected research clusters. The red cluster was centered on “nonhuman” and “probiotics” and was associated with “inflammation” and “microglia,” focusing on molecular mechanisms related to post-SCI inflammatory and immune responses. The green cluster was centered on “SCI” and linked to “human,” “complications,” and “urinary tract infection,” corresponding to studies on post-injury clinical characteristics and complication management. The blue cluster was organized around “neuroinflammation” and “animal” and linked to the “gut-brain axis” and “metabolism,” focusing primarily on SCI animal models and regulatory mechanisms of the gut-neural axis. Together, these three clusters comprehensively depict the research pathway from injury model construction and elucidation of pathological mechanisms to clinical translation.

#### 3.6.2 Thematic term analysis

Based on keyword co-occurrence analysis, Bibliometrix was further used to generate a thematic strategic diagram and analyze temporal thematic evolution, thereby systematically revealing the overall structure, dynamic characteristics, and evolutionary patterns of research themes in the field of SCI and the gut microbiota.

**Figure 8:**
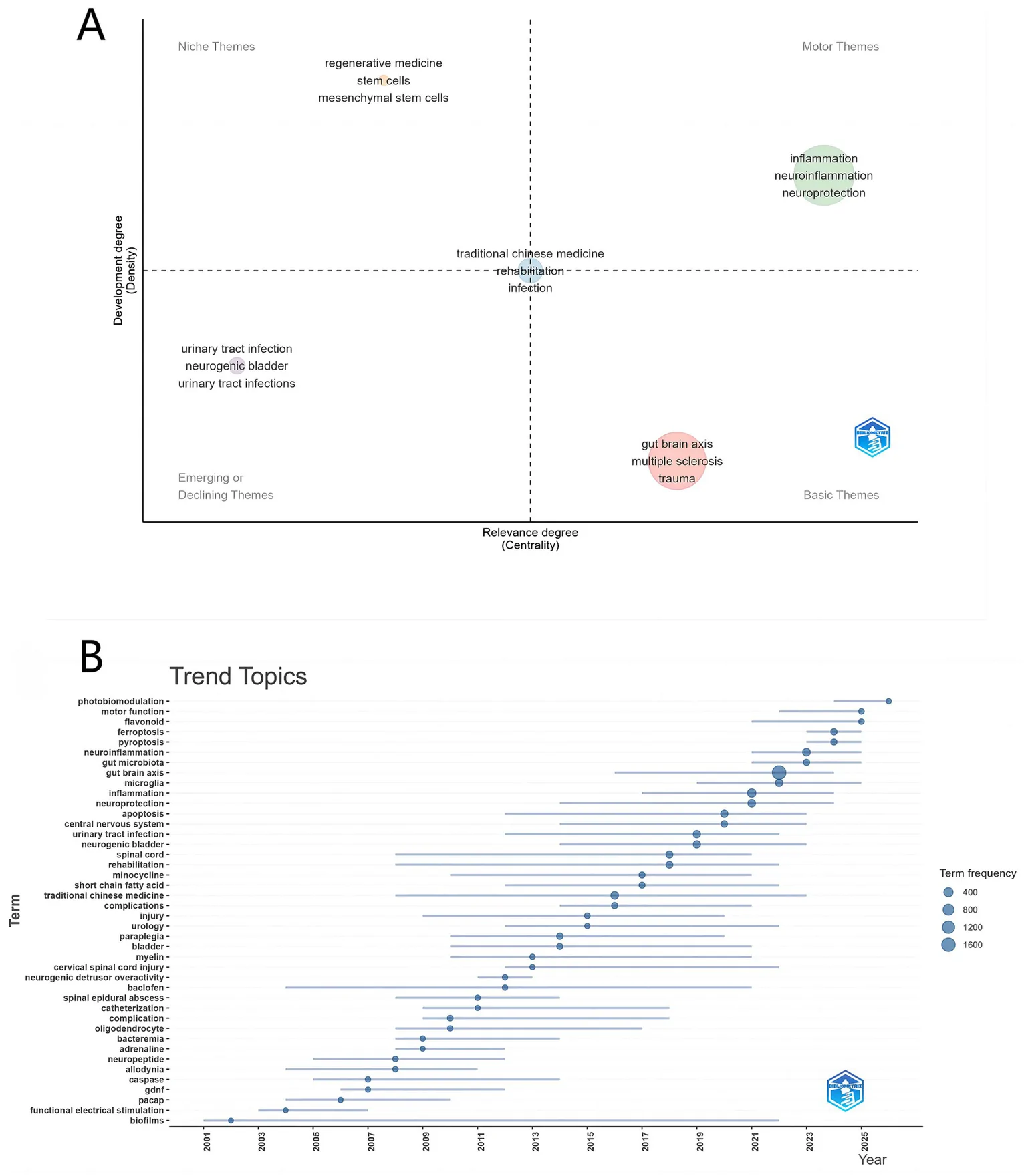
Strategic thematic and evolutionary trend analysis of keywords in research on spinal cord injury and the gut microbiota. **(A)** Strategic thematic map. The horizontal axis represents thematic centrality, reflecting the degree of relevance and overall influence of a theme within the field, whereas the vertical axis represents thematic density, reflecting the developmental maturity of the theme. The four quadrants correspond to motor themes (upper-right), basic themes (lower-right), niche themes (upper-left), and emerging or declining themes (lower-left). **(B)** Thematic evolution trend map, showing the temporal distribution and changes in research intensity of each theme from 2001 to 2025. Node size represents the total frequency of keyword occurrence, and the length of the horizontal line segment corresponds to the duration of research activity for that theme.

The thematic strategic diagram based on the centrality-density model (Figure 8A) revealed the research structure, dynamic characteristics, and developmental potential of this field. In the basic themes quadrant, themes such as “gut-brain axis,” “rehabilitation,” and “infection” showed high centrality, reflecting the long-standing core focus of the field on fundamental pathology and clinical rehabilitation after injury. In the motor themes quadrant, themes such as “inflammation,” “neuroinflammation,” and “neuroprotection” demonstrated both high centrality and rapid development, indicating that research on post-injury inflammatory mechanisms and neuroprotective strategies is currently highly active. In the niche themes quadrant, themes such as “regenerative medicine,” “stem cells,” and “mesenchymal stem cells” showed high density but low centrality, suggesting that the field has entered a phase of in-depth exploration of specific mechanisms and that such themes may become future growth points. The emerging or declining themes quadrant, which included topics such as “urinary tract infection” and “neurogenic bladder,” corresponded to specialized studies on SCI-specific complications and may represent potentially emerging directions that warrant further exploration.

The thematic trend analysis over time (Figure 8B) identified research hotspots across different periods and clearly reflected the evolution of research frontiers in this field. During 2001–2010, “baclofen,” “catheterization,” and “functional electrical stimulation” emerged as early hotspots, laying the groundwork for symptomatic treatment and basic management of SCI-related complications. Since 2010, themes such as “rehabilitation,” “traditional Chinese medicine,” and “complications” have continued to emerge, highlighting in-depth exploration of post-injury rehabilitation interventions and whole-course management. Since 2018, “photobiomodulation,” “flavonoids,” “ferroptosis,” “gut microbiota,” and the “gut-brain axis” have shown explosive growth and have become the core frontier directions in the field. These findings indicate a gradual shift in research focus—from early descriptions of pathological phenotypes and symptomatic management to investigations of underlying mechanisms and the development of targeted interventions strategies. Future development will likely move toward in-depth interpretation of molecular mechanisms and integrated application of multiple technologies.

## 4 Discussion

Using CiteSpace, VOSviewer, and Bibliometrix, this study conducted a systematic bibliometric analysis of 4,857 publications related to SCI and the gut microbiota indexed in the WoSCC and Scopus databases from January 2000 to March 2026. Through visualized multidimensional analyses of publication trends, collaboration networks among countries, institutions, and authors, journal distribution characteristics, co-citation networks, and keyword evolution patterns, this study comprehensively revealed the global research landscape, knowledge structure, and hotspot evolution of this field over the past 26 years. Overall, research on the bidirectional regulation between SCI and the gut microbiota has become an important interdisciplinary direction at the interface of neuroscience, rehabilitation medicine, and microbiology, with the research focus gradually shifting from early descriptive correlation studies to mechanistic exploration and clinical translation.

However, bibliometric findings should be interpreted critically, particularly when assessing the relationship between publication output and international influence. Although China ranked first in publication output, contributing 26.8% of all publications, this finding should be interpreted with caution rather than being regarded as evidence of dominant international influence. As shown in Table 1, China exhibited a very low proportion of multiple-country publications (MCP = 11, MCP% = 0.8%), indicating that the vast majority of publications were completed through domestic collaborations. Conversely, countries such as the United Kingdom (MCP% = 8.5%) and Canada (MCP% = 5.0%) demonstrated substantially higher levels of international collaboration despite producing fewer publications. This discrepancy suggests that publication quantity and international collaboration do not necessarily develop in parallel.

The collaboration networks shown in Figures 3A,B further support this observation. Although China and the United States were the two largest contributors and occupied the largest nodes in the collaboration network, international collaborations were concentrated among a relatively limited number of countries. In particular, the United States maintained broader collaborative ties with Europe, Canada, and Australia, whereas collaborations involving China appeared to be more regionally concentrated. Therefore, China’s leading publication output may partly reflect strong domestic research activity and sustained national investment in SCI and gut microbiota research. However, it may also indicate relatively limited international integration.

A similar pattern was observed at the institutional level (Figure 3C). Highly productive Chinese institutions, including Capital Medical University and Wenzhou Medical University, formed relatively localized collaborative clusters, whereas institutions from the United States, such as the Veterans Health Administration, the University of Washington, and Northwestern University, occupied more central positions within international institutional collaboration networks. These findings suggest that future research should not only emphasize publication output but also strengthen multicenter international collaboration, data sharing, and standardized clinical studies to improve the global impact and translational value of this field.

### 4.1 Analysis of highly cited literature and citation burst trends

Analysis of highly cited literature and citation bursts helps clarify the knowledge base of a field and delineate its developmental trajectory. The co-citation analysis in this study showed that the study by Kigerl KA, published in The Journal of Experimental Medicine in 2016, ranked first with 105 total citations and exhibited the highest citation burst strength (15.66) during 2018–2021, making it a landmark foundational study in this field (Kigerl et al., 2016). This study was the first to systematically demonstrate the detrimental effect of gut microbiota dysbiosis on neurological recovery after SCI, preliminarily establishing the theoretical framework of bidirectional regulation along the “gut-spinal cord axis” and suggesting that the microbiota-immune-neural repair axis is an important regulatory pathway in the pathological progression of SCI. At the same time, the locomotor function evaluation system established by Basso et al. (1995) and the seminal review published by McDonald and Sadowsky (2002) together constitute important methodological and theoretical foundations in this field. Furthermore, a series of studies by Gungor, Zhang, Jing, and their colleagues further enriched the understanding of bidirectional regulation between the gut microbiota and SCI (Gungor et al., 2016; Zhang et al., 2018; Jing et al., 2019), gradually forming a stable core knowledge system.

The co-citation cluster timeline and citation burst analyses further indicated that the research hotspots in this field show clear stage-specific evolutionary characteristics. During 1995–2010, research focused on the establishment of locomotor function assessment systems for SCI and the management of complications such as urinary tract infection, representing a stage of clinical and foundational accumulation. During 2010–2016, the research focus gradually shifted toward core pathological mechanisms such as post-injury inflammatory responses and neural injury-related signaling pathways, laying a solid mechanistic foundation for subsequent interdisciplinary research. Since 2016, gut microbiota-related themes have become the most active research direction in this field, with the citation burst strength of related literature continuing to rise and the research momentum persisting through 2026, indicating that interdisciplinary research on the gut microbiota and SCI has become the core frontier of the field.

Although current studies have preliminarily established the theoretical framework of the “gut-spinal cord axis,” many important scientific questions remain unresolved. First, the causal relationship between gut microbiota dysbiosis and the pathological progression of SCI has not yet been fully elucidated. Existing studies remain dominated by correlational analyses, making it difficult to determine whether specific microbial alterations drive pathological progression or merely secondary changes after injury. Second, the multi-pathway interactive mechanisms through which the gut microbiota regulates SCI require further in-depth investigation. Most existing studies have focused on single pathways, with insufficient attention given to the synergistic interactions among immune, metabolic, and neuroendocrine pathways, as well as to mechanistic heterogeneity across different injury stages and comorbid states. Third, substantial barriers remain in translating basic research into clinical practice. Although interventions such as probiotics and fecal microbiota transplantation have shown certain therapeutic effects in animal experiments, their colonization stability, interindividual response variability, and long-term safety in clinical settings still require further validation through large-sample, high-quality clinical studies.

### 4.2 Research hotspots and trends based on keywords

The keyword strategic diagram further reflected the developmental stages and future potential of different research themes. “Inflammation,” “neuroinflammation,” and “neuroprotection,” located in the motor themes quadrant with high centrality and high density, represent the most active and influential hotspots in the current field. “Gut-brain axis,” “rehabilitation,” and “infection,” located in the basic themes quadrant with high centrality and low density, constitute the core directions supporting the sustained development of the field. “Regenerative medicine,” “stem cells,” and “mesenchymal stem cells,” located in the niche themes quadrant with low centrality and high density, suggest substantial developmental potential. Themes related to SCI-specific complications, such as “urinary tract infection” and “neurogenic bladder,” located in the emerging or declining themes quadrant with low centrality and low density, currently receive relatively limited attention but still possess important clinical value and considerable scope for further investigation.

However, keyword co-occurrence and strategic diagram analyses also have inherent interpretive limitations. The prominence of terms such as “gut-brain axis,” “neuroinflammation,” “probiotics,” and “ferroptosis” indicates that these topics have attracted increasing research attention. However, it does not prove that these mechanisms are causally established in SCI or that related interventions are clinically effective. Furthermore, high-frequency keywords may be influenced by terminology preferences, indexing rules, and recent publication trends. Therefore, the hotspots identified in this study should be regarded as research priorities and hypothesis-generating directions rather than definitive mechanistic conclusions.

### 4.3 Core mechanisms by which the gut microbiota participates in the pathological process of SCI

Combined with the hotspot themes identified in this study and evidence from previous research, the gut microbiota appears to participate in the pathophysiological process of SCI throughout its course via multiple interconnected pathways, including microbial metabolic regulation, immune-inflammatory responses, and maintenance of barrier function. Importantly, the following mechanistic pathways should be understood as literature-derived hypotheses based on primary experimental studies and bibliometric hotspot analysis.

Potential association between gut microbiota dysbiosis and the pathological progression of SCI.

The keyword co-occurrence analysis in this study showed that “inflammation,” “gut microbiota,” and “oxidative stress” are core themes in this field, indicating that gut microbiota dysbiosis and inflammatory secondary injury have attracted substantial research attention in SCI-related microbiota studies. However, these bibliometric findings should be interpreted as indicators of research focus rather than direct evidence of causality.

Previous studies have reported that structural disturbances of the gut microbiota can occur as early as the acute phase of SCI (Jogia and Ruitenberg, 2020). Acute traumatic SCI may disrupt the spinal-autonomic gut pathway, thereby causing persistent gut microbiota dysbiosis and intestinal barrier damage. In turn, microbial dysbiosis has been hypothesized to aggravate secondary neural injury and functional impairment and increase infection risk by modulating systemic immune-inflammatory responses and altering the profile of microbial metabolites.

Furthermore, gut microbiota dysbiosis may contribute to disruption of host immune homeostasis by exacerbating imbalances in Treg/Th17 and Treg17/Teff17 differentiation and by disturbing the secretion of pro-inflammatory and anti-inflammatory cytokines, thereby amplifying peripheral and central immune-inflammatory responses and aggravating inflammatory infiltration and myelin injury (Lin et al., 2021). Meanwhile, microbial dysbiosis can also lead to impaired intestinal metabolic function, resulting in decreased levels of beneficial metabolites such as short-chain fatty acids and abnormal accumulation of neurotoxic metabolites, thereby exacerbating blood-spinal cord barrier disruption, neuronal apoptosis, and neurological deficits (Zhong et al., 2025).

Therefore, the role of gut microbiota dysbiosis in SCI progression should be considered a literature-derived mechanistic hypothesis supported by preclinical evidence, rather than a direct conclusion established by the present bibliometric analysis.

#### 4.3.1 Potential mediating role of short-chain fatty acids in SCI repair

The keyword statistics in this study showed that “short-chain fatty acids” had both high frequency of occurrence and strong node link strength, indicating that short-chain fatty acids represent a major research hotspot in studies of SCI and the gut microbiota. Nevertheless, keyword frequency and link strength reflect research attention and co-occurrence intensity, rather than proving that short-chain fatty acids are causal mediators of SCI repair.

Short-chain fatty acids are major beneficial metabolites produced by gut microbial fermentation of dietary fiber, primarily including acetate, propionate, and butyrate. Existing experimental studies suggest that their potential roles in SCI repair may be reflected in three aspects. First, by regulating the “gut-spinal cord-immune” axis, they may promote the differentiation of regulatory T cells toward an anti-inflammatory phenotype and stimulate IL-10 secretion, thereby suppressing excessive release of pro-inflammatory cytokines and attenuating central inflammatory responses mediated by microglial activation and astrocyte proliferation (Liu et al., 2023).

Second, by upregulating the expression of tight junction proteins in both the intestinal barrier and the blood-spinal cord barrier, they may enhance barrier integrity, block infiltration of peripheral inflammatory mediators into the central nervous system, and help restore the abundance of short-chain fatty acid-producing bacteria as well as gut microecological homeostasis (Rinderknecht et al., 2026). Third, by promoting neurotrophic factor expression, axonal regeneration, and motor neuron survival, they may reduce tissue injury, oxidative stress, and regulated cell death, thereby improving neurological recovery (Jing et al., 2023).

Thus, short-chain fatty acids should be presented as candidate microbiota-derived mediators and hypothesis-generating targets for SCI repair, requiring further causal validation through controlled mechanistic studies and clinical investigations.

#### 4.3.2 Potential neuroprotective effects of probiotics through targeted modulation of the gut microbiota

The high-frequency keyword analysis in this study identified “probiotics” as an important hotspot in clinical translational research.

Existing studies indicate that exogenous probiotic supplementation can restore gut microecological homeostasis, recover the abundance of short-chain fatty acid-producing bacteria, and upregulate the expression of intestinal tight junction proteins, thereby reducing endotoxemia and the initiation of peripheral inflammation (Trunz et al., 2026). At the same time, probiotics have been reported to inhibit activation of the TLR/NF-κB inflammatory signaling pathway within the injured spinal cord, modulate the activation phenotypes of microglia and astrocytes, promote the secretion of anti-inflammatory factors, and suppress the release of pro-inflammatory mediators, thereby creating a favorable neuroprotective microenvironment at the injury site, reducing neuronal apoptosis, promoting axonal regeneration, and improving motor function (Ileninová et al., 2025).

Overall, probiotic intervention has shown considerable neuroprotective potential in basic research, and related clinical studies also suggest potential value in preventing gastrointestinal complications in certain high-risk patients with SCI (Wong et al., 2024). However, current evidence remains limited, and its mechanisms of action, target populations, optimal strains and doses, colonization stability, and long-term safety still require further validation.

This result indicates increasing research interest in microbiota-targeted interventions, but it does not by itself demonstrate the therapeutic efficacy of probiotics in SCI. Accordingly, probiotics should be regarded as promising microbiota-targeted intervention candidates rather than established clinical therapies for SCI.

#### 4.3.3 Potential roles of other microbial metabolites and microbiota reconstruction strategies

In addition to short-chain fatty acids, other microbial metabolites and microbiota reconstruction strategies have been proposed as promising directions based on emerging experimental evidence. Studies have shown that the abundance of Limosilactobacillus reuteri declines significantly after SCI, and supplementation with *L. reuteri* DSM 17938 can promote tryptophan metabolism toward beneficial products such as indole-3-carboxaldehyde, activate AhR signaling, enhance tight junction protein expression, and inhibit microglial M1 polarization as well as the production of inflammatory mediators including IL-6, IL-1β, and TNF-*α*, thereby attenuating neuroinflammation and promoting functional recovery (Cen et al., 2026).

Another study demonstrated that the gut microbiota-derived tryptophan metabolite indole-3-propionic acid can suppress inflammatory activation of astrocytes through the AhR/NF-κB/MAPK axis, reduce glial scar formation, promote neuronal survival, and improve long-term motor function (Yu et al., 2026). Meanwhile, fecal microbiota transplantation, as a microbiota reconstruction strategy, can restore post-injury gut microbial diversity and compositional homeostasis, attenuate inflammatory responses, reduce extracellular matrix deposition and glial scar formation, and promote neuronal survival and axonal regeneration (Xie et al., 2025).

These findings suggest that interventions targeting the tryptophan metabolite-AhR signaling axis and microecological reconstruction represent important emerging directions beyond short-chain fatty acids. However, these signaling-axis statements are derived from primary experimental studies. They should be interpreted as mechanistic hypotheses summarized from the literature, rather than findings directly generated by the present bibliometric analysis. Further studies are required to determine whether these pathways are causal, reproducible across SCI models, and clinically translatable.

#### 4.3.4 Potential role of traditional Chinese medicine in SCI repair through regulation of the gut microbiota

In recent years, research on TCM has shown marked citation bursts and increasing publication output in the field of SCI, suggesting that interventions combining TCM with gut microbiota regulation and targeting novel forms of regulated cell death are gradually becoming important frontier directions in this area. Existing studies indicate that TCM may participate in SCI repair by modulating the gut microbiota and related metabolic pathways (Liu et al., 2024; Lu et al., 2020).

To enhance the clinical translational relevance of this topic, TCM-related interventions in the context of SCI should be considered a heterogeneous group of therapeutic strategies, including non-pharmacological therapies, bioactive natural compounds, and classical herbal formulas. These interventions may exert protective effects through gut microbiota remodeling, microbial metabolite regulation, intestinal barrier protection, immune-inflammatory modulation, and improvement of host metabolic homeostasis.

Electroacupuncture has been reported to improve intestinal transit function and colonic morphology in SCI rats, remodel altered microbial composition, and reverse the downregulation of the colonic 5-HT system, suggesting that its beneficial effects may be associated with regulation of the “microbiota-metabolite-5-HT” axis (Cheng et al., 2022). Moxibustion may alleviate post-SCI dysbiosis, reduce inflammatory cytokine levels, regulate macrophage polarization, and attenuate apoptosis, thereby contributing to motor function recovery (Zhang S. et al., 2022). Ursolic acid, as a representative TCM-derived bioactive compound, can optimize gut microbiota composition in mice with SCI, ameliorate metabolic disturbances, suppress inflammatory responses, and promote the expression of synapse-related proteins (Rong et al., 2022). Furthermore, Buyang Huanwu Decoction, a classical traditional Chinese herbal formula, has recently been reported to promote neurorepair after SCI by regulating the *Lactobacillus johnsonii*–indole-3-lactic acid–AhR–PI3K/Akt axis, providing further evidence that herbal formulas may influence SCI repair through microbiota-derived metabolites and host signaling pathways (Dong et al., 2026).

Overall, the role of TCM in SCI is not limited to local neuroprotection; it may also reshape the gut microecological environment, improve metabolic dysregulation, and modulate inflammatory responses, thereby forming a synergistic regulatory network between the gut microbiota and host repair processes. However, current evidence remains predominantly derived from animal experiments, and its mechanisms of action and translational clinical value still require further validation. Through a cascade involving “regulation of the gut microbiota-improvement of metabolic phenotype-suppression of neuroinflammation,” TCM may exert neuroprotective effects in a multitarget and holistic manner, offering a novel perspective on targeting the gut-spinal cord axis in SCI (Luo et al., 2024).

Nevertheless, these findings should be interpreted as literature-derived hypotheses rather than direct mechanistic conclusions from the present bibliometric analysis. Future studies should further identify the specific microbial taxa, microbial metabolites, and host signaling pathways affected by different TCM interventions. Furthermore, standardized intervention protocols, dose–response evaluation, safety assessment, and high-quality clinical trials are required before TCM-related microbiota modulation can be translated into clinical practice for patients with SCI.

### 4.4 Strengths and limitations

The strength of this study lies in its systematic bibliometric analysis of 4,857 publications in the emerging interdisciplinary field of SCI and the gut microbiota from 2000 to 2026. By incorporating both the WoSCC and Scopus databases, this study achieved a large sample size and a long time span, thereby broadly covering the developmental process of the field from its inception to its rapid expansion. Furthermore, by integrating three mainstream visualization tools, namely VOSviewer, CiteSpace, and Bibliometrix, this study conducted a comprehensive analysis from multiple dimensions, including publication trends, collaboration networks among countries and institutions, author and journal characteristics, co-citation networks, keyword co-occurrence, and temporal evolution, thereby systematically delineating the developmental trajectory, research hotspots, and emerging trends of this field and providing data support and directional guidance for subsequent basic research and clinical translation.

This study also has several limitations. First, to ensure data quality and compatibility with analytical software, only English-language original articles and reviews were included, and non-English publications were excluded, which may have led to underestimation of research output from some non-English-speaking countries. Second, although the search strategy was carefully designed and rigorously checked, some degree of retrieval bias may still exist, and relevant studies that did not include the core search terms in their titles or abstracts may have been missed. Third, this study included only records from the WoSCC and Scopus databases, excluding PubMed. Although PubMed does not provide complete citation metadata required for some citation-based bibliometric and co-citation analyses, its exclusion may still have introduced coverage bias, particularly for biomedical and clinically oriented studies. Therefore, the exclusion of PubMed should be regarded as a limitation on completeness of the present study rather than solely as a quality-control decision. Fourth, author name disambiguation is an inherent challenge in bibliometric analysis. Although duplicated or inconsistent author records identified during the revision process were manually checked and corrected as far as possible, variations in author names, initials, affiliations, and database indexing formats may still have affected author productivity ranking and collaboration network construction. Fifth, because the literature search was conducted up to March 25, 2026, the 2026 publication data represent only a partial year. Therefore, the apparent decrease in annual publication output in 2026 should not be interpreted as a true decline in research activity. This partial-year effect may influence the interpretation of recent publication trends and growth patterns. Finally, although bibliometric methods can reveal developmental characteristics and research trends at a macroscopic level, they are limited in their ability to assess in depth the methodological quality, rigor of experimental design, causal relationships, and translational value of individual studies. Future work should therefore combine systematic reviews, meta-analyses, experimental validation, and multicenter clinical studies to further validate the evidence base for the core research directions.

### 4.5 Future research directions

Based on the present findings, future research on SCI and the gut microbiota may be advanced in four major directions. First, greater emphasis should be placed on disease subtype stratification and precision research. Existing studies have primarily focused on traumatic SCI, with insufficient attention to the heterogeneity of microbial characteristics in non-traumatic SCI, different injury levels, and different injury severities. More refined stratified analyses are therefore needed to support individualized microbiota-based interventions. Second, mechanistic studies should integrate multi-omics technologies. By combining metagenomics, metabolomics, transcriptomics, and other omics approaches, microbiota-host interaction networks can be constructed to identify key molecular targets and signaling pathways underlying bidirectional regulation along the gut-spinal cord axis. Third, causal inference and clinical translation should be strengthened. Approaches such as Mendelian randomization and germ-free animal models may help clarify causal relationships between specific microbial taxa and SCI, whereas large-scale, multicenter randomized controlled trials are needed to verify the safety and efficacy of targeted microbiota-based interventions. Fourth, international collaboration and data sharing should be further promoted. The present findings indicate that deep transnational collaboration remains relatively limited in this field; therefore, future efforts should focus on strengthening multicenter collaborative innovation and data integration to promote high-quality development of the field.

In addition to microbiota-centered interventions, future translational research should also integrate advances from regenerative medicine, cell engineering, biomaterials, mitochondrial therapy, and immune microenvironment regulation. Recent studies have suggested that functionalized mesenchymal stem cells and mitochondrial transplantation/transfer may represent promising therapeutic strategies for SCI repair by improving neural regeneration, mitochondrial homeostasis, and the post-injury repair microenvironment (Jin et al., 2026; Xiong et al., 2025). Biomaterial–stem cell interactions may also provide important design principles for constructing scaffold-based or delivery-system-based regenerative strategies, which could be further combined with microbiota-targeted interventions in future studies (Shao et al., 2022). In parallel, bibliometric studies on macrophages in SCI and on tendon-derived stem cells have demonstrated the value of knowledge mapping in identifying neuroimmune and stem-cell-related research hotspots, thereby providing methodological and translational references for the present field (Shang et al., 2023; Zhang et al., 2024). Moreover, studies from adjacent biomedical and spinal degenerative fields have highlighted the potential relevance of oxidative stress-related epitranscriptomic regulation, Wnt/*β*-catenin signaling, and angiogenesis-related microenvironment remodeling, which may provide conceptual references for future mechanistic studies on SCI repair and microbiota–host interactions (Ruan et al., 2022; Zhang Z. et al., 2022; Wang et al., 2024).

## 5 Conclusion

Using bibliometric methods, this study systematically analyzed the global research landscape, knowledge structure, and developmental trends in the field of SCI and the gut microbiota from 2000 to 2026. The results showed that publication output in this field has increased steadily over the past 26 years and that this area has become an important interdisciplinary direction spanning neuroscience, rehabilitation medicine, and microbiology. China and the United States were the major contributing countries, while institutions such as Capital Medical University and Wenzhou Medical University represented important research forces in this field, and several relatively stable research teams have already emerged worldwide. This study identified the major research hotspots in this field, including the bidirectional regulatory mechanisms between the gut microbiota and SCI mediated by the gut-spinal cord axis; the mediating roles of microbial metabolites such as short-chain fatty acids; mechanisms of neuroinflammation and immune regulation, and gut microbiota-targeted intervention strategies. Among these, gut microbiota-mediated regulation of ferroptosis and other novel forms of regulated cell death, synergistic therapy combining stem cells with microbiota modulation, and the neuroprotective effects of TCM targeting the gut microbiota represent frontier directions that merit sustained attention. With the development of precision medicine and multi-omics technologies, future studies should further strengthen international collaboration, promote the integration of basic research with clinical rehabilitation, elucidate the key mechanisms by which the gut microbiota regulates SCI repair, and explore safer and more effective individualized intervention strategies, thereby providing new perspectives for improving neurological recovery and quality of life in patients with SCI.

## Data Availability

The original contributions presented in the study are included in the article/Supplementary material, further inquiries can be directed to the corresponding author.

## References

[ref1] Baas J. Schotten M. Plume A. Côté G. Karimi R. (2020). Scopus as a curated, high-quality bibliometric data source for academic research in quantitative science studies. Quant. Sci. Stud. 1, 377–386. doi: 10.1162/qss_a_00019

[ref2] Basso D. M. Beattie M. S. Bresnahan J. C. (1995). A sensitive and reliable locomotor rating scale for open field testing in rats. J. Neurotrauma 12, 1–21. 7783230 10.1089/neu.1995.12.1

[ref3] Basso D. M. Fisher L. C. Anderson A. J. Jakeman L. B. McTigue D. M. Popovich P. G. (2006). Basso mouse scale for locomotion detects differences in recovery after spinal cord injury in five common mouse strains. J. Neurotrauma 23, 635–659. doi: 10.1089/neu.2006.23.635, 16689667

[ref4] Beck K. D. Nguyen H. X. Galvan M. D. Salazar D. L. Woodruff T. M. Anderson A. J. (2010). Quantitative analysis of cellular inflammation after traumatic spinal cord injury: evidence for a multiphasic inflammatory response in the acute to chronic environment. Brain 133, 433–447. doi: 10.1093/brain/awp322, 20085927 PMC2858013

[ref5] Birkle C. Pendlebury D. A. Schnell J. Adams J. (2020). Web of science as a data source for research on scientific and scholarly activity. Quant. Sci. Stud. 1, 363–376. doi: 10.1162/qss_a_00018

[ref6] Cen Q. Cui Y. Feng J. Zhu L. Wei J. Wang L. . (2026). Limosilactobacillus reuteri DSM17938 attenuates neuroinflammatory responses after spinal cord injury by modulating tryptophan metabolism. Probiotics Antimicrob. Proteins 18, 583–601. doi: 10.1007/s12602-025-10545-y, 40281335 PMC12999684

[ref7] Chen Y. Zhou J. Wang L. (2021). Role and mechanism of gut microbiota in human disease. Front. Cell. Infect. Microbiol. 11:625913. doi: 10.3389/fcimb.2021.625913, 33816335 PMC8010197

[ref8] Cheng J. Li W. Wang Y. Cao Q. Ni Y. Zhang W. . (2022). Electroacupuncture modulates the intestinal microecology to improve intestinal motility in spinal cord injury rats. Microb. Biotechnol. 15, 862–873. doi: 10.1111/1751-7915.13968, 34797954 PMC8913878

[ref9] Dong J. Cao Y. Chen X. Xie T. Zhang X. Zhao Q. . (2026). Buyang Huanwu decoction promotes neurorepair after spinal cord injury through a *Lactobacillus johnsonii*–indole-3-lactic acid–AhR–PI3K/Akt axis. Chin. Med. 21:129. doi: 10.1186/s13020-026-01408-x, 42098851 PMC13154660

[ref10] Elliott C. Kreydin E. Crew J. Shem K. (2025). The current epidemiology of urinary incontinence and urinary tract infections after spinal cord injury-a model systems spinal cord injury examination (2016–2021). J. Clin. Med. 14:1434. doi: 10.3390/jcm14051434, 40094844 PMC11899757

[ref11] Esclarín De Ruz A. García Leoni E. Herruzo Cabrera R. (2000). Epidemiology and risk factors for urinary tract infection in patients with spinal cord injury. J. Urol. 164, 1285–1289. doi: 10.1016/S0022-5347(05)67157-1 10992382

[ref12] Gungor B. Adiguzel E. Gursel I. Yilmaz B. Gursel M. (2016). Intestinal microbiota in patients with spinal cord injury. PLoS One 11:e0145878. doi: 10.1371/journal.pone.0145878, 26752409 PMC4709077

[ref13] Hamilton A. M. Blackmer-Raynolds L. Li Y. Kelly S. D. Kebede N. Williams A. E. . (2024). Diet-microbiome interactions promote enteric nervous system resilience following spinal cord injury. NPJ Biofilms Microbiomes 10:75. doi: 10.1038/s41522-024-00556-y, 39209925 PMC11362535

[ref14] Hu X. Xu W. Ren Y. Wang Z. He X. Huang R. . (2023). Spinal cord injury: molecular mechanisms and therapeutic interventions. Signal Transduct. Target. Ther. 8:245. doi: 10.1038/s41392-023-01477-6, 37357239 PMC10291001

[ref15] Ileninová M. Bimbová K. K. Kisucká A. Bačová M. Mudroňová D. Kuruc T. . (2025). Early pro- and anti-inflammatory immune response induced by probiotic treatment after Th9 compression: the interplay between spinal cord and jejunum. Eur. J. Neurosci. 61:e70087. doi: 10.1111/ejn.70087, 40172540

[ref16] Jamali F. Mousavi S. Homayouni-Rad A. Meshkini A. Alikhah H. Houshyar J. . (2025). Exploring innovative approaches for managing spinal cord injury: a comprehensive review of promising probiotics and postbiotics. Probiotics Antimicrob. Proteins 17, 2112–2132. doi: 10.1007/s12602-025-10513-6, 40232596

[ref17] Jin S. T. Ma Y. G. Zhang S. O. (2026). Current status and future prospects of functionalized mesenchymal stem cells for the treatment of spinal cord injury. J. Orthop. Translat. 58:101125. doi: 10.1016/j.jot.2026.101125, 42110951 PMC13157060

[ref18] Jing Y. Yang D. Bai F. Wang Q. Zhang C. Yan Y. . (2023). Spinal cord injury-induced gut dysbiosis influences neurological recovery partly through short-chain fatty acids. NPJ Biofilms Microbiomes 9:99. doi: 10.1038/s41522-023-00466-5, 38092763 PMC10719379

[ref19] Jing Y. Yang D. Bai F. Zhang C. Qin C. Li D. . (2019). Melatonin treatment alleviates spinal cord injury-induced gut dysbiosis in mice. J. Neurotrauma 36, 2646–2664. doi: 10.1089/neu.2018.6012, 30693824

[ref20] Jogia T. Ruitenberg M. J. (2020). Traumatic spinal cord injury and the gut microbiota: current insights and future challenges. Front. Immunol. 11:704. doi: 10.3389/fimmu.2020.00704, 32528463 PMC7247863

[ref21] Kigerl K. A. Hall J. C. Wang L. Mo X. Yu Z. Popovich P. G. (2016). Gut dysbiosis impairs recovery after spinal cord injury. J. Exp. Med. 213, 2603–2620. doi: 10.1084/jem.20151345, 27810921 PMC5110012

[ref22] Lin X. Liu Y. Ma L. Ma X. Shen L. Ma X. . (2021). Constipation induced gut microbiota dysbiosis exacerbates experimental autoimmune encephalomyelitis in C57BL/6 mice. J. Transl. Med. 19:317. doi: 10.1186/s12967-021-02995-z, 34301274 PMC8306367

[ref23] Liu P. Liu M. Xi D. Bai Y. Ma R. Mo Y. . (2023). Short-chain fatty acids ameliorate spinal cord injury recovery by regulating the balance of regulatory T cells and effector IL-17^+^ γδ T cells. J. Zhejiang Univ. Sci. B 24, 312–325. doi: 10.1631/jzus.B2200417, 37056207 PMC10106403

[ref24] Liu G. Pei Z. Bai H. Huo L. Deng B. Jiang S. . (2024). Biomaterial-mediated delivery of traditional Chinese medicine ingredients for spinal cord injury: a systematic review. Front. Pharmacol. 15:1461708. doi: 10.3389/fphar.2024.1461708, 39545067 PMC11560789

[ref25] Lu Y. Yang J. Wang X. Ma Z. Li S. Liu Z. . (2020). Research progress in use of traditional Chinese medicine for treatment of spinal cord injury. Biomed. Pharmacother. 127:110136. doi: 10.1016/j.biopha.2020.110136, 32335299

[ref26] Luo T. Che Q. Guo Z. Song T. Zhao J. Xu D. (2024). Modulatory effects of traditional Chinese medicines on gut microbiota and the microbiota-gut-X axis. Front. Pharmacol. 15:1442854. doi: 10.3389/fphar.2024.1442854, 39444598 PMC11497133

[ref27] McDonald J. W. Sadowsky C. (2002). Spinal-cord injury. Lancet 359, 417–425. doi: 10.1016/S0140-6736(02)07603-1, 11844532

[ref28] Mincic A. M. Antal M. Filip L. Miere D. (2024). Modulation of gut microbiome in the treatment of neurodegenerative diseases: a systematic review. Clin. Nutr. 43, 1832–1849. doi: 10.1016/j.clnu.2024.05.036, 38878554

[ref29] Morimoto T. Kobayashi T. Kakiuchi T. Esaki M. Tsukamoto M. Yoshihara T. . (2023). Gut-spine axis: a possible correlation between gut microbiota and spinal degenerative diseases. Front. Microbiol. 14:1290858. doi: 10.3389/fmicb.2023.1290858, 37965563 PMC10641865

[ref30] Neurath M. F. Artis D. Becker C. (2025). The intestinal barrier: a pivotal role in health, inflammation, and cancer. Lancet Gastroenterol. Hepatol. 10, 573–592. doi: 10.1016/S2468-1253(24)00390-X, 40086468

[ref31] Ninkov A. Frank J. R. Maggio L. A. (2022). Bibliometrics: methods for studying academic publishing. Perspect. Med. Educ. 11, 173–176. doi: 10.1007/s40037-021-00695-4, 34914027 PMC9240160

[ref32] O'Connor G. Jeffrey E. Madorma D. Marcillo A. Abreu M. T. Deo S. K. . (2018). Investigation of microbiota alterations and intestinal inflammation post-spinal cord injury in rat model. J. Neurotrauma 35, 2159–2166. doi: 10.1089/neu.2017.5349, 29566601 PMC6119224

[ref33] Ohara T. E. Hsiao E. Y. (2025). Microbiota-neuroepithelial signalling across the gut-brain axis. Nat. Rev. Microbiol. 23, 371–384. doi: 10.1038/s41579-024-01136-9, 39743581

[ref34] Orr M. B. Gensel J. C. (2018). Spinal cord injury scarring and inflammation: therapies targeting glial and inflammatory responses. Neurotherapeutics 15, 541–553. doi: 10.1007/s13311-018-0631-6, 29717413 PMC6095779

[ref35] Rinderknecht S. Bertolo A. Valido E. Stojic S. Wong S. Farkas G. J. . (2026). The role of short-chain fatty acids in spinal cord injury: a systematic review of human and animal evidence. J. Spinal Cord Med., 1–14. doi: 10.1080/10790268.2025.2607831, 41711677 PMC13591990

[ref36] Rong Z. J. Cai H. H. Wang H. Liu G. H. Zhang Z. W. Chen M. . (2022). Ursolic acid ameliorates spinal cord injury in mice by regulating gut microbiota and metabolic changes. Front. Cell. Neurosci. 16:872935. doi: 10.3389/fncel.2022.872935, 35602557 PMC9115468

[ref37] Ruan L. Zhang S. Chen X. Liang W. Xie Q. (2022). Role of anti-angiogenic factors in the pathogenesis of breast cancer: a review of therapeutic potential. Pathol. Res. Pract. 236:153956. doi: 10.1016/j.prp.2022.153956, 35700578

[ref38] Shang J. Jiang C. Cai J. Chen Z. Jin S. Wang F. . (2023). Knowledge mapping of macrophage in spinal cord injury: a bibliometric analysis. World Neurosurg. 180, e183–e197. doi: 10.1016/j.wneu.2023.09.022, 37714458

[ref39] Shao R. Dong Y. Zhang S. Wu X. Huang X. Sun B. . (2022). State of the art of bone biomaterials and their interactions with stem cells: current state and future directions. Biotechnol. J. 17:e2100074. doi: 10.1002/biot.202100074, 35073451

[ref40] Sun L. Peng C. Joosten E. Cheung C. W. Tan F. Jiang W. . (2021). Spinal cord stimulation and treatment of peripheral or central neuropathic pain: mechanisms and clinical application. Neural Plast. 2021:5607898. doi: 10.1155/2021/5607898, 34721569 PMC8553441

[ref41] Trunz J. Schmandt C. Hertig-Godeschalk A. Glisic M. Stoyanov J. Perret C. (2026). Probiotic and prebiotic supplementation for gastrointestinal discomfort in chronic spinal cord injury (PRO-GIDSCI): a randomized controlled crossover trial protocol. Methods Protoc. 9:14. doi: 10.3390/mps9010014, 41562992 PMC12821591

[ref42] Wang F. Wang Y. Zhang S. Pu M. Zhou P. (2024). YTHDF2-dependent m6A modification of FOXO3 mRNA mediates TIMP1 expression and contributes to intervertebral disc degeneration following ROS stimulation. Cell. Mol. Life Sci. 81:477. doi: 10.1007/s00018-024-05503-w, 39625652 PMC11615171

[ref43] Wong S. Hirani S. P. Forbes A. Kumar N. Hariharan R. O’Driscoll J. . (2024). *Lactobacillus casei* Shirota probiotic drinks reduce antibiotic associated diarrhoea in patients with spinal cord injuries who regularly consume proton pump inhibitors: a subgroup analysis of the ECLISP multicentre RCT. Spinal Cord 62, 255–263. doi: 10.1038/s41393-024-00983-w, 38519563 PMC11176055

[ref44] Xie H. Zhang H. Zhou L. Chen J. Yao S. He Q. . (2025). Fecal microbiota transplantation promotes functional recovery in mice with spinal cord injury by modulating the spinal cord microenvironment. J. Transl. Med. 23:210. doi: 10.1186/s12967-025-06232-9, 39979990 PMC11843963

[ref45] Xiong X. Zhou C. Yu Y. Xie Q. Xia L. Li Q. . (2025). Mitochondrial transplantation/transfer: promising therapeutic strategies for spinal cord injury. J. Orthop. Translat. 52, 441–450. doi: 10.1016/j.jot.2025.04.017, 40485848 PMC12145523

[ref46] Yu D. Kang J. Jiang W. Zhang C. Zhao X. Zhao Z. . (2026). Indole-3-propionic acid inhibits astrocyte inflammation and promotes motor function recovery after spinal cord injury via the AhR/NF-κB/MAPK axis. Neuropharmacology 289:110855. doi: 10.1016/j.neuropharm.2026.110855, 41663028

[ref47] Zhang S. He L. Shang J. Chen L. Xu Y. Chen X. . (2022). Carvacrol suppresses human osteosarcoma cells via the Wnt/β-catenin signaling pathway. Anti Cancer Agents Med. Chem. 22, 1714–1722. doi: 10.2174/1871520621666210901111932, 34488595

[ref48] Zhang S. Shang J. Gu Z. Gu X. Wang F. Hu X. . (2024). Global research trends and hotspots on tendon-derived stem cell: a bibliometric visualization study. Front. Bioeng. Biotechnol. 11:1327027. doi: 10.3389/fbioe.2023.1327027, 38260747 PMC10801434

[ref49] Zhang Z. Sui R. Ge L. Xia D. (2022). Moxibustion exhibits therapeutic effects on spinal cord injury via modulating microbiota dysbiosis and macrophage polarization. Aging (Albany NY) 14, 5800–5811. doi: 10.18632/aging.204184, 35876627 PMC9365548

[ref50] Zhang C. Zhang W. Zhang J. Jing Y. Yang M. Du L. . (2018). Gut microbiota dysbiosis in male patients with chronic traumatic complete spinal cord injury. J. Transl. Med. 16:353. doi: 10.1186/s12967-018-1735-9, 30545398 PMC6293533

[ref51] Zhong Z. Fan F. Lv J. Wang Z. Wang B. Deng C. . (2025). Changes of potential shorty-chain fatty acids producing bacteria in the gut of patients with spinal cord injury: a systematic review and meta-analysis. Front. Microbiol. 16:1483794. doi: 10.3389/fmicb.2025.1483794, 40083777 PMC11905530

