# Supplementary material for: Bibliometric analysis of research on spinal cord injury and the gut microbiota
Source: Front Microbiol. 2026 Aug 6;17:1852992. doi: 10.3389/fmicb.2026.1852992 (PMC13490134; doi:10.3389/fmicb.2026.1852992)
Supplement: Supplementary file 2 [file Data_Sheet_2.pdf]

Supplementary Material S2
Complete Search Strategy

Overview

Item	Description
**Databases**	Web of Science Core Collection (WoSCC) and Scopus
**Retrieval date**	March 25, 2026
**Time span**	January 1, 2000 to March 25, 2026
**Language**	English
**Document types**	Original articles and review articles
**Initial records**	WoSCC: 718; Scopus: 5,840; total: 6,558
**Screening outcome**	1,200 duplicate records were removed; 5,358 records remained; 501 non-articles/non-reviews were excluded; 4,857 publications were included in the bibliometric analysis.

Note. The search strategy was designed to identify publications related to spinal cord injury and the gut microbiota. All searches were completed on the same day to minimize retrieval bias caused by daily database updates.

1. Web of Science Core Collection

Item	Description
**Database**	Web of Science Core Collection
**Search field**	Topic (TS)
**Retrieval date**	March 25, 2026
**Timespan**	January 1, 2000 to March 25, 2026
**Document type filter**	Article OR Review
**Language filter**	English
**Records retrieved**	718

Search query:

```
TS = (
  (
    "spinal cord injur*" OR "spinal cord traumat*" OR "spinal cord contus*"
    OR "spinal cord transect*" OR "spinal cord lacerat*"
    OR "spinal cord lesion*" OR "traumatic myelopath*"
    OR "post-traumatic myelopath*" OR "acute spinal cord injury"
    OR "chronic spinal cord injury" OR "incomplete spinal cord injury"
    OR ("SCI" AND "spinal cord")
  )
  AND
  (
    ((gut OR intestin* OR enteric OR gastrointestin* OR colonic)
    AND (microbiot* OR microbiome* OR microflora OR flora OR bacteria
    OR microbial OR microorganism* OR commensal*))
    OR "Gut Microbiota" OR "Intestinal Microbiota"
    OR "Gut Microbiome" OR "Intestinal Microbiome"
    OR "Gut Flora" OR "Intestinal Flora"
    OR dysbiosis OR dysbacteriosis
    OR "gut dysbiosis" OR "intestinal dysbiosis"
    OR "flora imbalance" OR "gut-brain axis" OR "brain-gut axis"
    OR "microbiota-gut-brain axis"
    OR ("GBA" AND "gut-brain axis")
```

```
    OR "gut-spinal axis" OR "spinal-gut axis"
    OR "gut-spinal cord axis"
    OR "microbiota-gut-spinal axis"
    OR "microbiome-gut-spinal cord axis"
    OR ((gut OR intestin* OR enteric OR "gut microbiota")
    AND (neuro* OR CNS OR spinal OR "spinal cord")
    AND (axis OR interaction OR crosstalk OR "cross talk"
    OR signaling OR regulation))
    OR probiotic* OR prebiotic* OR synbiotic* OR postbiotic*
    OR antibiotic* OR "fecal microbiota transplantation"
    OR ("FMT" AND "fecal microbiota transplantation")
    OR "gut barrier" OR "intestinal barrier"
    OR "gut permeability" OR "intestinal permeability"
    OR "microbial metabolite*"
  )
)
```

2. Scopus

Item	Description
**Database**	Scopus
**Search field**	Title, Abstract, and Keywords (TITLE-ABS-KEY)
**Retrieval date**	March 25, 2026
**Publication years**	2000–2026
**Document type filter**	Article OR Review
**Language filter**	English
**Records retrieved**	5,840

Search query:

```
TITLE-ABS-KEY (
  "spinal cord injur*" OR "spinal cord traumat*" OR "spinal cord contus*"
  OR "spinal cord transect*" OR "spinal cord lacerat*"
  OR "spinal cord lesion*" OR "traumatic myelopath*"
  OR "post-traumatic myelopath*" OR "acute spinal cord injury"
  OR "chronic spinal cord injury" OR "incomplete spinal cord injury"
  OR ("SCI" AND "spinal cord")
)
AND
TITLE-ABS-KEY (
  ((gut OR intestin* OR enteric OR gastrointestin* OR colonic)
  AND (microbiot* OR microbiome* OR microflora OR flora OR bacteria
  OR microbial OR microorganism* OR commensal*))
  OR "Gut Microbiota" OR "Intestinal Microbiota"
  OR "Gut Microbiome" OR "Intestinal Microbiome"
  OR "Gut Flora" OR "Intestinal Flora"
  OR dysbiosis OR dysbacteriosis
  OR "gut dysbiosis" OR "intestinal dysbiosis"
  OR "flora imbalance" OR "gut-brain axis" OR "brain-gut axis"
  OR "microbiota-gut-brain axis"
  OR ("GBA" AND "gut-brain axis")
  OR "gut-spinal axis" OR "spinal-gut axis"
  OR "gut-spinal cord axis"
  OR "microbiota-gut-spinal axis"
  OR "microbiome-gut-spinal cord axis"
  OR ((gut OR intestin* OR enteric OR "gut microbiota")
  AND (neuro* OR CNS OR spinal OR "spinal cord")
  AND (axis OR interaction OR crosstalk OR "cross talk"
  OR signaling OR regulation))
  OR probiotic* OR prebiotic* OR synbiotic* OR postbiotic*
```

```
    OR antibiotic* OR "fecal microbiota transplantation"
    OR ("FMT" AND "fecal microbiota transplantation")
    OR "gut barrier" OR "intestinal barrier"
    OR "gut permeability" OR "intestinal permeability"
    OR "microbial metabolite*"
  )
  AND PUBYEAR > 1999
  AND PUBYEAR < 2027
  AND (LIMIT-TO (DOCTYPE, "ar") OR LIMIT-TO (DOCTYPE, "re"))
  AND LIMIT-TO (LANGUAGE, "English")
```

Additional note for Scopus. The final Scopus search was restricted to journal articles and reviews published in English between 2000 and 2026. Source types outside the scope of the review, such as book chapters and conference-related records, were excluded during database filtering and screening according to the eligibility criteria.

3. Screening and inclusion summary

The literature screening process is summarized in Figure 1 of the manuscript. Briefly, 6,558 records were retrieved from WoSCC and Scopus. After removal of 1,200 duplicate records, 5,358 records remained for screening. A total of 501 records were excluded because they were not original articles or review articles. Finally, 4,857 publications were included in the bibliometric analysis, including 3,134 original articles and 1,723 review articles.
